# Five-country analysis of geographic and dietary drivers of gut microbiome composition and genomic variation

**DOI:** 10.1038/s41467-026-77928-9

**Published:** 2026-09-21

**Authors:** Lora Khatib, Renee Oles, Alejandra Ríos Hernández, Tyler Myers, Jianshu Zhao, Megha Kumar, Ana Laroya, Veronica Tolosa-Enguis, Nobuko Matsunami, Daisuke Suzuki, Nodoka Chiba, Katharine E. Gilbert, Promi Das, Jon G. Sanders, Lucas Patel, Nicole Litwin, Brent Nowinski, Amanda Birmingham, Sawyer Farmer, Caitriona Brennan, MacKenzie Bryant, Caitlin Tribelhorn, Karenina Sanders-Bodai, Charles Cowart, Oliver Nizet, Edgar A. Diaz, Patrick Veiga, Sudarshan A. Shetty, Jan Knol, Christophe Lay, Hana Koutnikova, Soline Chaumont, Julien Tap, Aurélie Cotillard, Muriel Derrien, Manolo Laiola, Antonio González, Armando R. Tovar, Nimbe Torres, Yolanda Sanz, Takuji Yamada, Daniel McDonald, Se Jin Song, Andrew Bartko, Rob Knight

**Affiliations:** 1 Department of Pediatrics, University of California San Diego, La Jolla, CA USA; 2 Neurosciences Graduate Program, University of California San Diego, La Jolla, CA USA; 3 Center for Microbiome Innovation, University of California San Diego, La Jolla, CA USA; 4 Department of Mathematics, University of California San Diego, La Jolla, CA USA; 5 Department of Cognitive Science, University of California San Diego, La Jolla, CA USA; 6 Microbiome Innovation in Nutrition & Health Research Unit, Institute of Agrochemistry and Food Technology, Spanish National Research Council (IATA-CSIC), Paterna-Valencia, Spain; 7 School of Life Science and Technology, Institute of Science Tokyo, Tokyo, Japan; 8 Gutz Analytics, Boulder, CO USA; 9 Bioinformatics and Systems Biology Program, University of California San Diego, La Jolla, CA USA; 10 Medical Scientist Training Program, University of California San Diego, La Jolla, CA USA; 11 Crohn’s & Colitis Foundation, New York, NY USA; 12 Division of Biological Sciences, University of California San Diego, La Jolla, CA USA; 13 Department of Chemical and Biomolecular Engineering, Johns Hopkins University, Baltimore, MD USA; 14 Department of Research & Health Promotion, San Ysidro Health, San Diego, CA USA; 15 Université Paris-Saclay, INRAE, MGP, Jouy-en-Josas, France; 16 Université Paris-Saclay, INRAE, AgroParisTech, UMR1319 MICALIS Institute, Jouy-en-Josas, France; 17 Danone Research & Innovation, Utrecht, the Netherlands; 18 Precision Nutrition D-Lab, Danone Global Research & Innovation Center, Singapore, Singapore; 19 Danone Research & Innovation, Gif sur Yvette, France; 20 VIB-KU Leuven Center for Microbiology, Leuven, Belgium; 21 Laboratory of Molecular Bacteriology, Department of Microbiology and Immunology, Rega Institute, KU Leuven, Leuven, Belgium; 22 Department of Nutritional Physiology, Instituto Nacional de Ciencias Medicas y Nutricion Salvador Zubiran, Mexico City, Mexico; 23 Shu Chien-Gene Lay Department of Bioengineering, University of California San Diego, La Jolla, CA USA; 24 Department of Computer Science and Engineering, University of California San Diego, La Jolla, CA USA; 25 Halıcıoğlu Data Science Institute, University of California San Diego, La Jolla, CA USA; 26 Hong Kong University of Science and Technology Jockey Club Institute for Advanced Study, Hong Kong, China

**Keywords:** Metagenomics, Microbiota

## Abstract

Human diet and lifestyle vary widely across cultures, shaping global diversity in gut microbiome composition and function. However, comparable cross-population studies have been limited by methodological heterogeneity. The Human Diets and Microbiome Initiative (THDMI) integrates standardized recruitment, dietary assessment, and shotgun metagenomics across 1,976 participants from the United States, United Kingdom, Spain, Mexico, and Japan to disentangle biological from technical variation. We observed pronounced geographic differences in nutrient intake, food consumption, and microbial composition, with both shared and country-specific features linked to diet. Sparse canonical correlation and machine-learning analyses revealed that microbiome–diet associations were largely population-specific, with limited cross-country generalization. Strain-level analyses further uncovered significant genomic differentiation of gut microbes, consistent with localized dietary and ecological differences. Together, these results show that while core microbial responses to healthy eating exist, the structure and function of the human gut microbiome varies strongly with culture, geography, and long-term dietary practices.

## Abstract (web summary)

In this study, authors show that human gut microbiome composition and microbial genomes vary strongly across five industrialized countries, with diet microbiome links that are partly shared but largely population-specific.

## Introduction

Patterns of human diet and lifestyle vary profoundly across the globe, shaped largely by geography, culture, and socioeconomic context, with genetic or ethnic factors contributing to a lesser extent^1^. These differences contribute to heterogeneity in dietary exposures, chronic disease risk, and health outcomes across populations. A growing body of work demonstrates that the gut microbiome mediates key effects of diet and environment on host physiology, influencing metabolic function, immune responses, and nutrient metabolism^2–5^. Yet, the extent to which diet-microbiome interactions are shared across cultures versus shaped by population-specific ecological and evolutionary pressures remains unresolved.

Early cross-population studies revealed striking geographic structuring of the gut microbiome, especially between drastically contrasting lifestyles, with agrarian or hunter-gatherer communities often exhibiting greater microbial diversity and distinct taxonomic and functional profiles compared to industrialized populations^6–10^. These differences are often attributed to dietary shifts from fiber-rich, minimally processed foods toward Westernized patterns high in refined grains, added sugars, and fats. Controlled dietary interventions further support a dominant role for diet, with extreme changes restructuring microbial communities within days, while modest modifications yield subtler responses^11–14^. Microbial signatures associated with immigration and acculturation additionally suggest that dietary convergence may coincide with loss of microbial diversity and functions linked to traditional diets^15^.

Despite these advancements, large-scale multi-country investigations with standardized microbiome profiling and harmonized diet and metadata collection remain rare^16,17^. Comparisons across independent cohorts are often confounded by technical variability (e.g., sampling, extraction, sequencing, and bioinformatics), which can obscure biological signals. Consequently, apparent cross-country differences may reflect methodological heterogeneity rather than genuine ecological divergence.

To mitigate these challenges, we present The Human Diets and Microbiome Initiative (THDMI), a coordinated multi-country study encompassing 1976 participants from five industrialized nations (United States (US), United Kingdom (UK), Japan, Spain, and Mexico). THDMI integrates standardized recruitment, biospecimen processing, shotgun metagenomic sequencing and analysis, and harmonized dietary and lifestyle assessments, enabling more comparable datasets across sites, while minimizing technical variability through standardized protocols. Using these data, we aimed to (i) characterize cross-country variation in demographic, dietary, and microbial features, and (ii) determine whether microbial associations with host and dietary factors are shared or population-specific. Our findings reveal both conserved and context-dependent host-microbiome-diet interactions, offering insight into how cultural and dietary practices may influence microbial ecology.

## Results

### Population characteristics

**Fig. 1:**
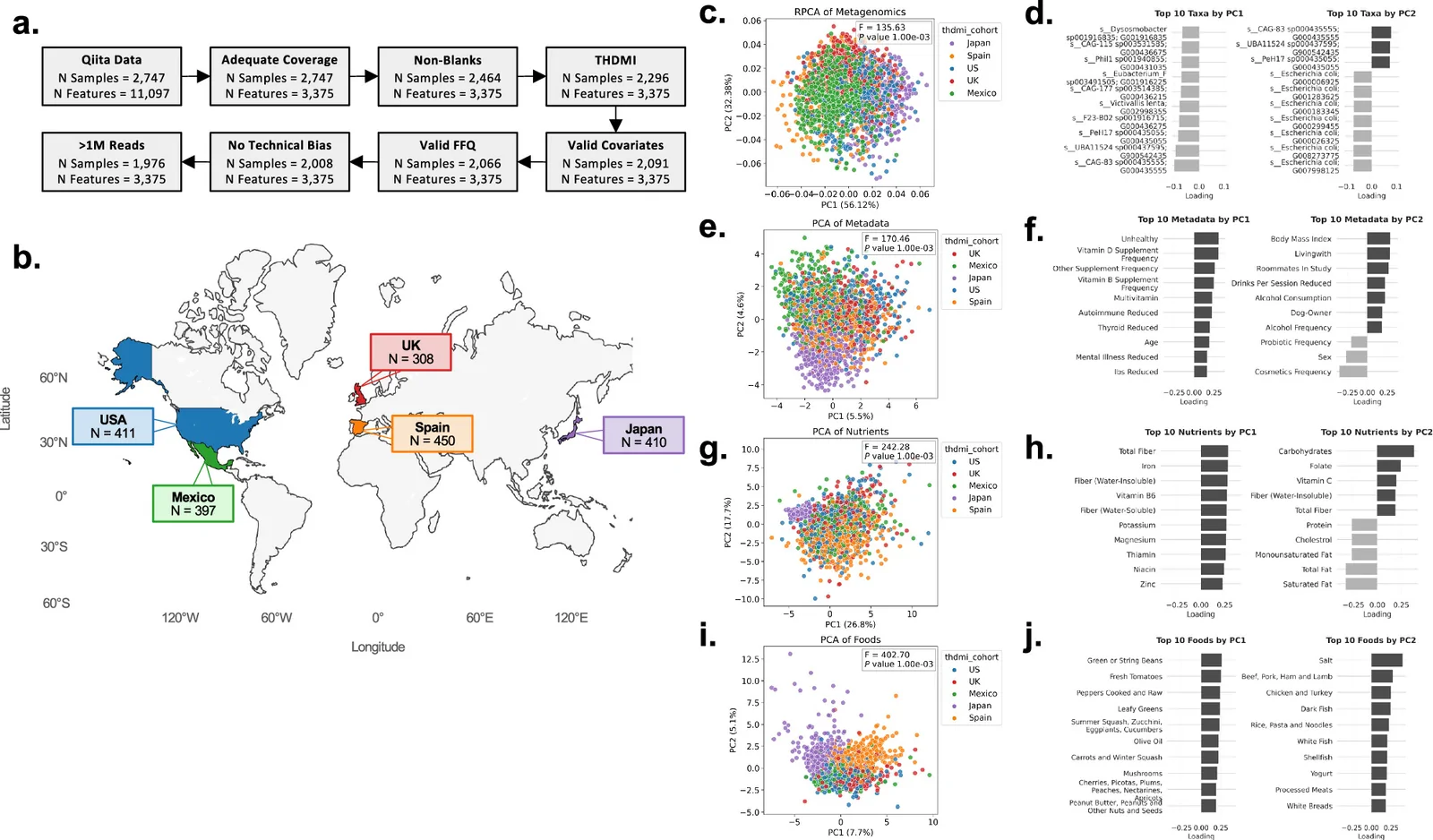
Population characteristics. **a** Starting with 2747 samples and 11,097 features from Qiita, successive filtering steps (including removal of low-coverage features, blank controls, misattributed samples, samples with invalid covariate or FFQ data, and those failing read-depth thresholds) yielded a final dataset of 1976 samples and 3375 features. **b** Geographic distribution of participants. The final THDMI cohort includes individuals from five countries: USA (*N* = 411), Mexico (*n* = 397), UK (*n* = 308), Spain (*n* = 450), and Japan (*n* = 410). Principal component analyses (PCAs; *n* = 1976). Variation across cohorts was visualized for metagenomic features (**c**, **d**), metadata (**e**, **f**), nutrients (**g**, **h**), and foods (**i**, **j**). (**c**, **e**, **g**, **i**): Scatterplots of the first two principal components colored by cohort. Differences in multivariate composition among countries were assessed using permutation-based PERMANOVA (999 permutations); pseudo-F statistics and exact permutation *P* values are shown in each panel. (**d**, **f**, **h**, **j**): the top 10 features driving Principal Component 1 (PC1) and Principal Component 2 (PC2), respectively. Bars represent PCA loading coefficients for the indicated principal component.

After quality control, the analytic cohort included 1976 participants across the US, Mexico, UK, Spain, and Japan (Fig. 1a, b). Demographic variables differed significantly across countries (Table 1), most prominently in cosmetics use (75% Japan vs. ≤ 20%; *P* < 0.001), body mass index (BMI) (25.7 Mexico vs. 21.7 Japan; *P* < 0.001), and age distributions (52.1 UK vs. 42.0 Mexico; *P* < 0.001). Notably, sequencing depth also differed across countries (*P* < 0.001). However, the potential contribution of these variables to observed clustering was evaluated in downstream analyses and did not explain the primary patterns of variation (see below and “Methods”).

**Table 1:** Demographics of participants

Parameter	Japan	Mexico	Spain	UK	US	Statistic	*Q*-value
No. of participants	410 (20.7%)	397 (20.1%)	450 (22.8%)	308 (15.6%)	411 (20.8%)		
Sequencing Depth	5825616.0 ± 301174.7	8319371.6 ± 3387466.9	10130394.9 ± 10001755.8	10198694.0 ± 7643289.0	15772525.0 ± 9313275.1	428.9	8.86e-91
Age	47.5 ± 9.4	42.0 ± 15.2	42.6 ± 6.6	52.1 ± 13.3	45.0 ± 15.2	160.0	4.84E-33
BMI	21.7 ± 3.6	25.7 ± 5.1	23.4 ± 4.8	25.1 ± 5.7	25.2 ± 5.3	204.5	2.05E-42
Sex: Female	354 (86.3%)	269 (67.8%)	374 (83.1%)	223 (72.4%)	262 (63.7%)	83.886	4.36E-17
Unhealthy	151 (36.8%)	212 (53.4%)	251 (55.8%)	195 (63.3%)	249 (60.6%)	66.958	1.24E-13
Bowel movement quality: normal	245 (61.3%)	301 (77.8%)	235 (55.6%)	219 (75.3%)	300 (76.5%)	74.516	3.60E-15
Antibiotic history: within the last month	38 (13.1%)	47 (19.0%)	38 (12.8%)	11 (4.3%)	11 (3.8%)	34.445	6.04E-07
Types of plants: more than 30	32 (14.8%)	17 (7.2%)	19 (8.8%)	53 (55.2%)	57 (32.0%)	61.227	1.78E-12
Types of plants: less than 10	184 (85.2%)	220 (92.8%)	197 (91.2%)	43 (44.8%)	121 (68.0%)	152.538	1.45E-31
Cosmetics frequency: daily	308 (75.1%)	50 (12.6%)	70 (15.6%)	61 (19.8%)	61 (14.8%)	580.531	2.53E-123
Exercise frequency: daily	33 (8.0%)	35 (8.8%)	40 (8.9%)	86 (27.9%)	79 (19.2%)	90.519	2.04E-18

Dietary assessments used country-specific validated FFQs, which were harmonized to shared food codes and standardized food groupings (see Source Data). Following harmonization, 29 nutrients and 60 aggregated food groups were retained. Nutrient and food intake varied significantly by country (Tables S1 and S2), with particularly large differences in dietary patterns consistent with regional staples (e.g., rice/pasta/noodles and sodium-rich foods).

To quantify cross-country structure across domains, we performed principal component analysis (PCA) on metadata, nutrients, and food groups, as well as robust PCA (RPCA) on metagenomic profiles (Fig. 1c–j). All domains demonstrated strong clustering by country (PERMANOVA *P* <.001). Microbial separation was driven largely by obligate anaerobes within *Clostridia* and multiple *Escherichia coli* operational genomic units (OGUs; Fig. 1d; see Fig. S1 for schematic). However, we were unable to determine whether *E. coli* OGUs represent distinct strains or reflect multimapping to a single strain. Metadata variables driving variation included clinical conditions, BMI, supplement use, and lifestyle factors such as cosmetic use and roommate status (Fig. 1f). Nutrients driving separation included fiber, iron, vitamin B6, carbohydrates, and fats (Fig. 1h). Important food drivers included green or string beans, tomatoes, salt, red meats, and poultry (Fig. 1j). Japan and Spain exhibited the greatest between-country dissimilarity across microbiome and diet profiles (Fig. S2).

To assess whether demographic and technical variables contributed to observed microbiome clustering, we performed PERMANOVA including cosmetics use, BMI, age, sex, and sequencing depth as covariates. Geographic differences remained highly significant (*F* = 191.3, *P* < 0.001), with country (*R*² = 0.28) explaining substantially more variation than cosmetics use (*R*² = 0.006), BMI (*R*² = 0.003), age (*R*² = 0.001), sex (*R*² = 0.002), or sequencing depth (*R*² = 0.005). Consistent results were observed in sensitivity analyses restricted to female participants (*F* = 107.6, *P* < 0.001) and to individuals with BMI in the normal range (*F* = 88.3, *P* < 0.001).

### Associations between the microbiome, metadata, nutrients, and foods

**Fig. 2:**
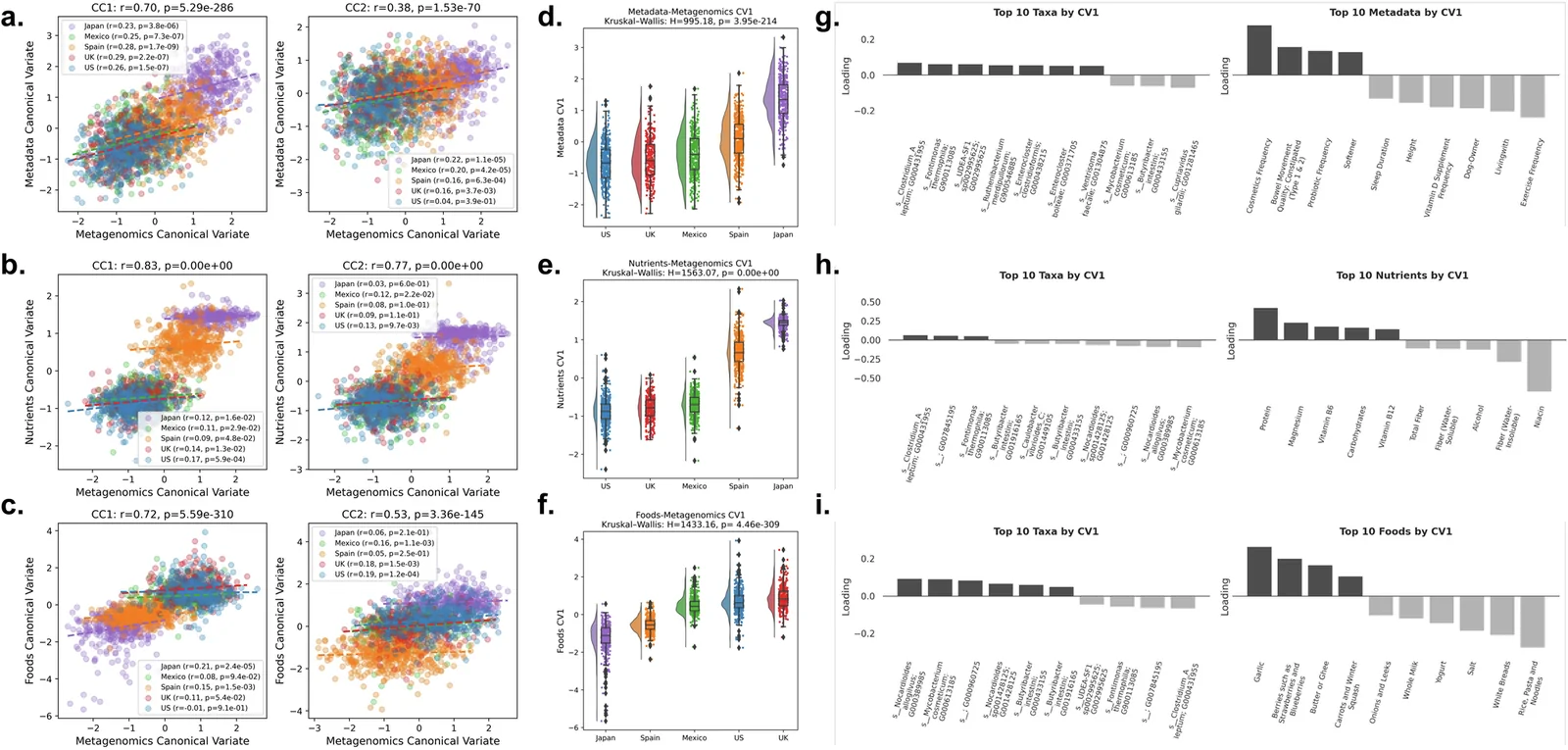
Sparse canonical correlation analysis (sCCA) of metagenomics with metadata, nutrients, and foods. Scatterplots of canonical variates (CVs) from sCCA across five countries, showing correlations between metagenomics and **a** metadata, **b** nutrients, and **c** foods. The left panels display Canonical Correlation 1 (CC1), whereas the right panels display Canonical Correlation 2 (CC2). Each point represents an individual, colored by country, with best-fit regression lines for each cohort. Pearson correlation coefficients (*r*) were calculated using two-sided Pearson correlation tests. Exact *P* values are shown for each country and for all participants (*n* = 1976). Raincloud plots consisting of violin plots overlaid with boxplots and individual observations of the first canonical variate (CV1) scores by country for **d** metadata, **e** nutrients, and **f** foods, illustrating cross-country distributional differences. In the boxplots, the center line represents the median, the box bounds represent the interquartile range (25th–75th percentiles), and the whiskers extend to the most extreme data points within 1.5× the interquartile range. Individual points are shown. For **f**, CV1 values were multiplied by −1 to facilitate direct visual comparison with **d**, **e**. Group differences were assessed using two-sided Kruskal–Wallis tests (H statistic). Exact *P* values are shown in each panel (*n* = 1976). **g**–**i** Barplots of the top 10 features with the largest absolute loadings on CV1 for each view. Positive and negative bars indicate direction of association. **g** Top metagenomic taxa and metadata variables, **h** top metagenomic taxa and nutrient variables, and **i** top metagenomic taxa and food variables.

Next, we performed sparse canonical correlation analysis (sCCA)^18^ to evaluate relationships between metagenomic profiles and host metadata, nutrient intake, and food consumption across cohorts (Fig. 2). sCCA revealed strong correlations between the microbiome and metadata (*r* = 0.70, *p* < 0.0001; Fig. 2a), nutrients (*r* = 0.83, *p* < 0.0001; Fig. 2b), and foods (*r* = 0.72, *p* < 0.0001; Fig. 2c), driven largely by between-country heterogeneity. Canonical variate 1 (CV1) further confirmed large cross-country differences for all domains (Kruskal–Wallis *P* < 0.0001; Fig. 2d–f), with Japan and Spain typically separating from Mexico, US, and UK.

Feature loadings highlighted microbial and host/diet drivers of canonical variates (Fig. 2g–i). Among microbes, OGUs such as *Clostridium_A leptum*, *Butribacter intestini*, *Fontimonas thermaphila*, and *Mycobacterium cosmeticum* showed strong contributions across all domains. Key metadata contributors included cosmetics use, constipation, probiotic use, and exercise. Diet contributors included protein, magnesium, niacin, and water-insoluble fiber, and foods such as garlic and rice/pasta/noodles.

Within-country sCCA revealed consistent correlations across domains (Figs. S3–S5). Overlap of top microbial OGUs across countries was limited. However, some OGUs such as *Enterocloster clostrioformis*, *Campylobacter_A concisus*, *Cloacibacillus evryensis*, *Acidaminococcus intestini*, and *Bacteroides_F pectinophilus* overlapped between two to three countries. Host metadata features most strongly associated with metagenomic profiles included exercise frequency, bowel movement quality, and age. Cholesterol was a top nutrient along with MUFA and fiber. Oranges, grapefruit, and tangerines were among overlapping foods correlated with metagenomic profiles. Motivated by this pattern, we tested whether *B. pectinophilus* is associated with pectin intake and observed positive associations in three countries with pectin data present (Fig. S5k; Ps < 0.05).

More broadly, many within-country associations reflected regionally variable or culturally salient foods, although exceptions suggested that frequency of intake alone does not fully explain association strength.

### Associations with the Healthy Eating Index differ across countries

Overall dietary patterns have been shown to exert a stronger influence on the gut microbiome than individual foods^19^. To capture these broader dietary patterns, we used the Healthy Eating Index (HEI) 2015^20^, which integrates food- and nutrient-based components of diet quality into a composite score for each participant based on Dietary Guidelines for Americans.

**Fig. 3:**
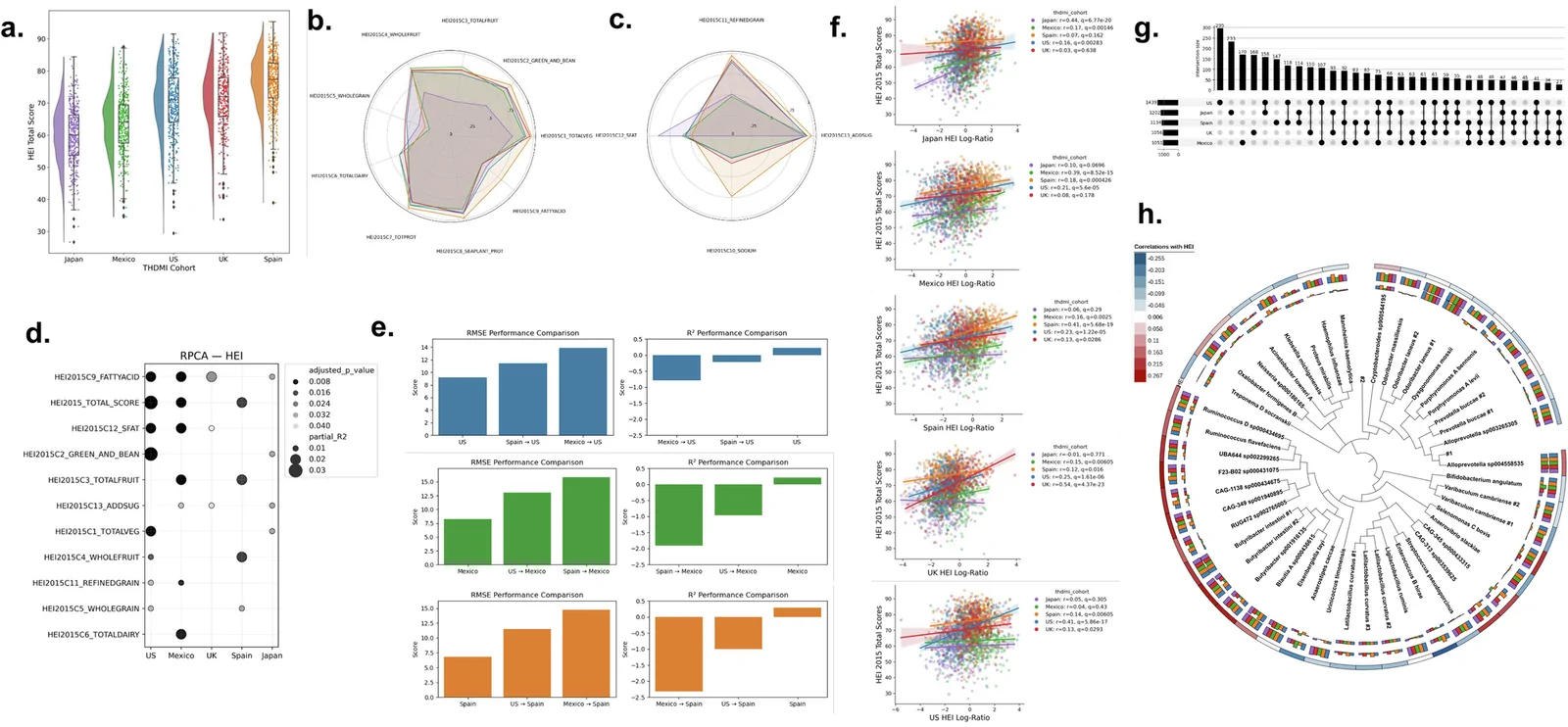
Healthy Eating Index (HEI) across countries. Healthy Eating Index (2015) scores were calculated for all participants. **a** Distribution of HEI total scores across the five THDMI cohorts. Boxplots show the median (center line), interquartile range (box), and whiskers extending to the most extreme data points within 1.5 × the interquartile range; individual observations are overlaid (*n* = 1976). **b**, **c** Radar plots of average HEI component scores by country. **d** Robust principal component analysis (RPCA) results linking HEI components to gut microbiome variation across countries. Associations between HEI components and microbiome composition were assessed using permutation-based PERMANOVA, adjusting for age, sex, and BMI. *P* values were corrected for multiple comparisons using the Benjamini–Hochberg false discovery rate (FDR), and adjusted *P* values are shown. Each circle represents a HEI component, with color denoting adjusted *p*-value, and circle size corresponding to the partial *R*² (effect size) of the association. **e** Bar plots showing Root Mean Square Error (RMSE) (left) and *R*² (right) from Elastic Net regressions trained on the HEI-2015 Total Score. Models were trained using 5-fold cross-validation within each country and then evaluated across countries. **f** Scatter plots show the relationship between HEI total scores and the log-ratio of positively to negatively associated taxa identified by BIRDMAn within each country. Each panel corresponds to a model trained in one country (Japan, Mexico, Spain, UK, US) and tested across all five THDMI cohorts. Lines represent the mean fitted regression, and shaded bands indicate the 95% confidence interval around the fitted mean. Points are colored by country. Spearman correlation coefficients (*r*) and associated *q*-values are shown. **g** UpSet plot showing overlap in HEI-associated taxa across countries. **h** Phylogenetic distribution of the 48 taxa associated with HEI total scores across five countries. Inner ring: Centered log-ratio–transformed abundance of each taxon, stratified by country. Middle ring: Prevalence of each taxon by country. Outer ring: Spearman correlations between taxa and HEI total scores. Correlation strength is represented by a continuous color scale (left), with positive correlations in red and negative correlations in blue. Zero or near-zero correlations are shown in white. Branch lengths were disabled during visualization to emphasize taxon relationships rather than evolutionary distances.

Total HEI scores differed substantially across countries (Fig. 3a; *F* = 204.69, *p* < 0.0001), with Spain exhibiting the highest (“healthiest”) average scores, followed by the UK, US, Mexico, and Japan. Across the “adequacy” components, those reflecting recommended food groups, Spain showed the highest scores for greens and beans, fatty acids (MUFA/PUFA), and seafood and plant proteins (Fig. 3b), whereas Japan showed the lowest scores for greens and beans, fruit, and whole grains. For the “moderation” components, which reflect foods to limit, Spain again performed best, reporting the lowest intake of sodium, refined grains, and added sugars, while Japan scored lowest (indicating higher intake) for sodium and refined grains (Fig. 3c). However, Japan reported lower saturated fat intake compared to the other countries.

To examine microbiome-diet relationships, we performed PERMANOVA analyses within each country, testing associations between metagenomic profiles and both HEI total and component scores, adjusting for age, sex, and BMI (Fig. 3d). No HEI component was significantly associated with microbiome composition across all five countries, confirming substantial cross-cultural differences in microbiome-diet relationships, even at higher food classification levels. Significant associations with the total HEI score, as well as several component scores, were observed in the US, Mexico, and Spain, whereas Japan and the UK showed limited associations.

We next evaluated whether countries with significant HEI-microbiome associations shared similar predictive microbial OGUs. Elastic Net regressors were trained within each country and then cross-applied to other countries (Fig. 3e). Both root mean square error (RMSE) and *R*² declined markedly when models trained in one country were tested on another, compared to within-country testing, suggesting that distinct microbial OGUs underlie diet-microbiome associations across populations. Interestingly, Spain and Mexico exhibited lower cross-country generalization compared to models involving the US, even though their microbiome profiles were more similar than to those of other countries (Fig. S2).

To test this further, we ran Bayesian Inferential Regression for Differential Microbiome Analysis (BIRDMAn)^21^ against the HEI total score within each country. For each country, we calculated the log ratio of positively associated OGUs to negatively associated OGUs and then computed Spearman correlations between these log ratios and the HEI total scores (Fig. 3f). This approach allowed us to evaluate whether OGUs associated with healthy eating overlapped across countries. Interestingly, several countries showed overlapping microbial associations. In Japan, OGUs associated with healthy eating were also significant in Mexico and the US, but not in Spain or the UK. In Mexico, similar OGUs were associated with diet quality in the US and Spain, but not in the UK or Japan. In Spain and the UK, overlapping OGUs were shared with all other countries except Japan. In the US, overlapping OGUs linked to healthy eating were shared primarily with Spain and the UK.

Lastly, we compared the number of individual microbial OGUs correlated with HEI across countries (Fig. 3g). The majority of OGUs associated with healthy eating were country-specific, providing further evidence towards strong geographic variation in microbiome-diet relationships. However, several OGUs overlapped across countries, with 48 OGUs shared across all five cohorts. Phylogenetic analysis of these shared OGUs revealed a distinct clade within the *Clostridia* class that was positively correlated with healthy eating and generally depleted in Mexico compared to other countries (Fig. 3h).

### Prevalence and associations of microbial OGUs across countries

We next examined the prevalence and associations of microbial OGUs across the five countries (Fig. S6). We tested for OGUs with ≥60% prevalence in one country and ≤10% prevalence in all others. Only three *Bacillus subtilis* OGUs met this criterion, all of which were found almost exclusively in Japan. Within Japan, these OGUs were consistently depleted in participants with thyroid conditions and enriched among those reporting daily multivitamin use. In addition, *B. subtilis* OGUs showed positive associations with several nutrients, including vitamin C, isoflavones, magnesium, potassium, and folate, and negative associations with cholesterol. All three genomes demonstrated strikingly elevated and continuous coverage within Japanese participants (Fig. S7A). In contrast, samples from the US, UK, Mexico, and Spain showed sparse and discontinuous coverage. To evaluate prior reports that *B. subtilis* is enriched in the Japanese gut microbiome due to natto consumption^22^, we examined the association between *B. subtilis* abundance and fermented foods among Japanese participants and observed a strong correlation with natto intake (Fig. S7B; *r* = 0.59, *p* = 4.9 × 10^−39^).

We next identified enriched OGUs, defined as those at least three times more prevalent in one country than in any other. This analysis yielded 48 enriched OGUs (Fig. S6). Although some showed correlations with metadata, nutrients, or foods, these OGUs generally exhibited low coverage and prevalence across samples (Table S3).

### Country-specific genomic differentiation of microbial species

To assess whether gut microbial species exhibit country-specific genomic structure, we assembled metagenome-assembled genomes (MAGs), quality-filtered at ≥80% completeness ≤10% contamination, and clustered at the species level (average nucleotide identity, ANI ≥ 95%). Species with ≥30 MAGs (*n* = 73) were retained. This threshold ensures adequate statistical power for PERMANOVA across five countries.

Because MAG recovery can be influenced by assembly success, sequencing depth, and genome characteristics, we also assessed species detection by mapping quality-controlled metagenomic reads back to the dereplicated MAG database. For each of the 73 species-level genome bins retained for genome-resolved analyses, we compared MAG recovery, breadth-based prevalence using a ≥40% genome-breadth threshold, and median mapped-read abundance across the US, UK, Spain, Mexico, and Japan cohorts (Fig. S8). Many retained species were broadly detectable by read mapping, although MAG recovery, prevalence, and abundance were not identical across cohorts. These results provide complementary biological context and clarify that the ≥30 MAG threshold was used as a statistical-power criterion rather than as a direct proxy for species prevalence.

**Fig. 4:**
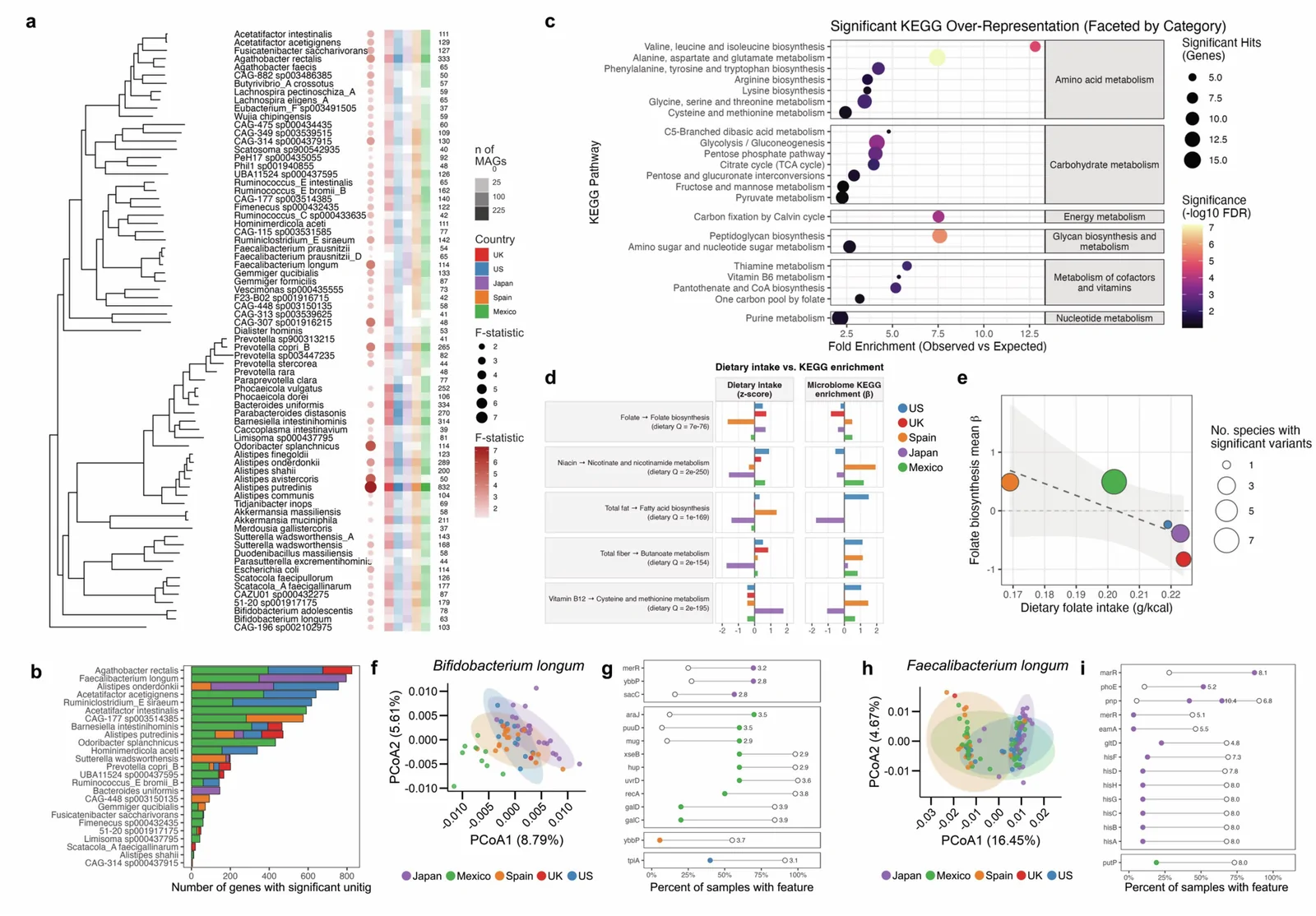
Country-specific genomic differentiation of microbial species. Metagenome-assembled genomes (MAGs) were quality-filtered (≥80% completeness, ≤10% contamination) and clustered at the species level (ANI ≥95%). For each species, pairwise genomic distances were computed using Mash. Pairwise Mash distance matrices were tested for country-level differences using permutation-based PERMANOVA (999 permutations; *n* = 1976). Pseudo-F statistics are shown for each species. **a** Phylogenetic distribution of representative genomes of species with ≥30 members. The adjacent F-statistic represents the significance of the Mash distance matrix by country using PERMANOVA. The number next to the heatmap represents number of MAGs per species. **b** Number of genes with significant geographically structured variants per species identified from unitig-based association testing. **c** KEGG pathway enrichment analysis of geographically structured genes across species. Each point represents a pathway; size indicates the number of contributing genes, and color represents statistical significance (–log₁₀ FDR). Pathways are grouped by functional category, including amino acid metabolism, carbohydrate metabolism, energy metabolism, glycan biosynthesis, cofactor and vitamin metabolism, and nucleotide metabolism. **d** Comparisons between dietary intake and microbiome functional enrichment across countries. Left panels show standardized dietary intake (*z*-scored), and right panels show corresponding microbiome KEGG pathway enrichment (*β* coefficients). Colors indicate country. **e** Relationship between dietary folate intake and microbial folate biosynthesis enrichment across countries. Each point represents a country; size reflects the number of species with significant variants, and the dashed line indicates the overall trend, with the shaded band depicting 95% confidence interval around the fitted mean. Within-species principal-coordinate analysis (PCoA) of Mash distances for two representative taxa: *Bifidobacterium longum* (**f**) and *Faecalibacterium longum* (**h**). Points are colored by country, with 95% confidence ellipses. Prevalence of top population-associated genes within *B. longum* (**g**) and *F. longum* (**i**). Points represent the proportion of genomes carrying each gene within a given country; colors indicate the focal country relative to others.

Pairwise genomic distances were computed using Mash (a MinHash-based method for fast estimation of genome-wide similarity)^23^ to quantify genomic relatedness. We used PERMANOVA to test whether Mash genomic distances were significantly structured by country of origin per species. Thirty-five species met significance after FDR correction (FDR < 0.05), demonstrating non-random clustering of genetic lineages by country (Fig. 4a). Several lineages within the *Lachnospiraceae* family and multiple *Prevotella* species showed consistently strong associations with country of origin.

To characterize the functional basis of this differentiation, we performed unitig-based genome-wide association analyses using pyseer^24^, mapping significant variants to genes and KEGG pathways. The number of geographically differentiated genes varied substantially across species (Fig. 4b). Enrichment analysis identified KEGG pathways significantly over-represented among geographically structured variants (FDR < 0.05), spanning carbohydrate metabolism, amino acid biosynthesis, cofactor and vitamin metabolism, and energy metabolism (Fig. 4c). Population-specific enrichment of metabolic functions was widespread, and within each population the magnitude and direction of enrichment varied among co-resident species rather than converging on identical loci, consistent with a distributed, polyfunctional microbial network (Fig. S9a).

We next asked whether geographically differentiated microbial pathways were associated with measured dietary variation across the five cohorts. Several nutrients showed coherent patterns with pathway enrichment, although often in opposite directions across countries (Fig. 4d). A notable example was dietary folate, which differed significantly across countries (Table S1; *Q* = 7.37 × 10⁻⁷⁶), with Spain (0.169 g/kcal) and Mexico (0.202 g/kcal) reporting the lowest intake and Japan (0.223 g/kcal) and the UK (0.224 g/kcal) the highest (Fig. 4d). Microbial folate biosynthesis gene enrichment inversely tracked this dietary gradient across all five countries (Fig. 4e): strains from Spain (*β* = +0.49, 3 species) and Mexico (*β* = +0.50, 7 species) showed enrichment, whereas strains from Japan (β = −0.39, 3 species) and the UK (*β* = −0.83, 2 species) showed depletion. This pattern suggests a potential reduction in reliance on microbial folate biosynthesis in populations with higher dietary folate intake, and greater retention of biosynthetic capacity in populations with lower intake.

Total fat intake also differed substantially across cohorts (*Q* = 1.44 × 10⁻¹⁶⁹), with Japan showing the lowest intake (0.032 g/kcal) and corresponding depletion of fatty acid biosynthesis signals (*β* = −1.75), whereas the US (0.042 g/kcal; *β* = +1.50) showed enrichment (Fig. 4d). Vitamin B12 intake was also higher in Japan (0.004 g/kcal) compared to the remaining cohorts (0.002 g/kcal; *Q* = 2.12 × 10⁻¹⁹⁵), and Japanese strains showed depletion of cysteine and methionine metabolism genes (*β* = −1.06, 4 species), whereas strains from Mexico, Spain, and the US showed enrichment (*β* = +0.67 to +1.48), consistent with a potential reduction in reliance on one-carbon metabolism pathways in a high-B12 dietary context (Fig. 4d). Across all nutrients, associations with microbiome functional pathways were highly heterogeneous, with several pathways exhibiting opposing directions of association across populations, indicating context-dependent microbial metabolic responses to diet (Fig. S9b).

To investigate species-level patterns, we selected two species with high MAG recovery across all five countries and significant PERMANOVA results for gene presence/absence analysis. In *Bifidobacterium longum*, principal-coordinate analysis of Mash distances revealed clear separation by country (PERMANOVA *F* = 2.24, *p* < 0.01; Fig. 4f). MAGs from Mexico exhibited a marked loss of galactose-metabolism genes (*galC*, *galD*), retained in >70 % of non-Mexican genomes but in <10 % of genomes from Mexico (Fig. 4g), consistent with the high prevalence of lactose intolerance in this population (31.1%)^9^. Conversely, *sacC*, associated with sucrose and fructan metabolism, was enriched in genomes from Japan. In *Faecalibacterium longum*, genomes from Japan were depleted of histidine-biosynthetic genes (*hisA–F*; Fig. 4h, i), whereas genomes from Mexico showed significant loss of *putP*, encoding a proline transporter.

Similar country-specific patterns were present across a diverse array of species (see Source Data). For example, *Prevotella copri_B* exhibited the strongest genomic differentiation in the Mexican cohort, driven primarily by variants in plant polysaccharide degradation genes (*xyl3A*, *uxaC*) and nucleotide biosynthesis. However, some species showed widespread lineage divergence rather than pathway-specific separation.

These findings suggest that the relationship between dietary intake and microbial functional output is strongly population-specific and may not generalize across geographic contexts. While we observe some links between measured dietary intake and microbial genomic variation, the majority of differentiated pathways, including amino acid biosynthesis, carbohydrate metabolism, and cofactor production, cannot be confidently attributed to specific dietary variables on the basis of cross-sectional data alone. These broader patterns of genomic differentiation may reflect species-level lineage divergence, microbe–microbe cross-feeding, host genetic factors, or other environmental variables not captured in this study.

## Discussion

This study represents one of the first single-study, cross-cultural analyses of diet, lifestyle, and the gut microbiome in industrialized societies. Leveraging shotgun metagenomics, lifestyle and dietary intake data, and genomic resolution of microbial lineages, we examined 1976 participants from the US, UK, Spain, Mexico, and Japan. We found pronounced differences across countries in metadata variables, dietary patterns, and microbiome composition. These differences were accompanied by country-specific and shared microbial signatures of diet, as well as clear genomic differentiation of microbial lineages, highlighting strong associations between cultural lifestyle, dietary practices, and the structure and function of the gut microbiome.

Aligned with prior cross-population studies^6–9^, we observed pronounced differences in microbiome composition and function, dietary intake, and lifestyle across countries. Several features driving this separation have been reported previously, such as a gradient toward lower BMI in Japan^25^ and higher intake of olive oil in Spain^26^. Interestingly, Spain and Mexico harbored the most taxonomically similar gut microbiome profiles among the cohorts. However, cross-country generalization between them was unexpectedly low when compared to the US. This suggests that while the overall community composition may reflect shared genetic ancestry or historical population structure, consistent with prior studies linking host genetics to microbial features^27,28^, the functional capacities of these communities may have diverged in response to distinct dietary patterns and lifestyles.

Further, we find substantial heterogeneity in microbiome-diet associations across countries. Sparse canonical correlation analysis revealed pronounced cross-country differences in microbiome-diet canonical structures, indicating distinct context-dependent relationships between dietary intake and microbial composition. However, it is not possible to fully disentangle true biological variation from technical bias arising from differences in FFQs and nutrient calculation methodologies. These findings highlight a critical need for global nutrient harmonization to enable more accurate cross-population comparisons and to advance our understanding of host-microbiome relationships.

Here, no HEI component was consistently associated with microbiome composition in all cohorts, and machine-learning models trained to predict diet quality in one country generally failed to transfer across populations. These findings further suggest that diet-microbiome relationships are context-dependent and vary across populations and highlight the need to consider alternative dietary scoring systems. It is also important to note that cross-country diet-microbiome comparisons assume sufficient overlap in detectable OGUs across populations, which may not always hold.

Importantly, however, one subset of microbial OGUs, largely within the class *Clostridia*, showed consistent associations with healthier eating patterns across countries, highlighting a potentially evolutionarily conserved microbial core responsive to diet quality. Yet, these findings largely challenge the assumption that universal microbiome markers of dietary health exist, and instead point to context-dependent microbial responses to diet. Cultural dietary substrates, culinary processing techniques, food availability, and habitual co-consumption patterns may contribute to population-specific nutritional ecotypes, underscoring the need for stratified, population-aware microbiome-targeted nutritional interventions.

Our strain-level analyses further highlight geography-linked microbial differences. Multiple species exhibited significant country-specific genomic differentiation, particularly within fiber-fermenting lineages such as *Prevotella* and *Lachnospiraceae*, extending prior observations in traditional versus Westernized populations^6–10^. Differential enrichment of carbohydrate-active enzymes and amino acid biosynthetic pathways across countries suggests that microbes may vary in association with not only host genetic or environmental factors, but also long-term population-level dietary exposures. These findings resonate with emerging evidence that fine-scale microbial population structure is consistent with localized ecological differentiation^29,30^.

Together, our results reveal that while core ecological principles may link diet and microbial function globally, the microbial signatures of dietary quality are not universal. However, several limitations warrant acknowledgment. As with most crowdsourced initiatives, our populations are enriched in women and individuals with relatively high diet quality. Further efforts are needed to capture more representative populations. Although using country-specific FFQs is methodologically appropriate, a potential bias arises from harmonizing these instruments to a single nutrient database and applying standardized HEI scoring across countries. In addition, our analyses relied primarily on food-group-level intake, which may reduce sensitivity to detect certain food-microbiome associations. Further, although standardized protocols were used across all sites, we cannot fully exclude residual technical variation, as mock community controls were not included. Finally, the analysis is cross-sectional, and causal mechanisms remain to be established. Observed associations may reflect unmeasured confounders or correlated environmental factors. Interventional studies across diverse dietary cultures will be critical for validating country-specific and shared microbial determinants of nutritional responsiveness.

In sum, this work provides one of the most comprehensive examinations to date of global diet-microbiome relationships across distinct populations, suggesting that while specific microbial signatures of diet quality are generic, microbiome-diet interactions may be shaped by culture, geography, and dietary patterns. As the field advances toward precision nutrition, these findings support a shift from universal microbiome dietary biomarkers to culturally informed, population-aware frameworks that capture the full breadth of human dietary ecology.

## Methods

### Sample collection

Adult participants were consented through the Microsetta Initiative platform from the US, UK, Mexico, Spain, and Japan. The study was approved by the University of California San Diego Institutional Review Board, under the University of California San Diego Human Research Protections Program (Protocol #141853). For the Mexico cohort, ethical approval was also obtained from the Comité de Ética en Investigación of the Instituto Nacional de Ciencias Médicas y Nutrición Salvador Zubirán (INCMNSZ) (Protocol #3853), because some recruitment occurred through in-person interactions at affiliated clinical sites. All participants provided written informed consent. For the other countries, additional IRB protocols were not required; however, we collaborated with local institutions to ensure compliance with country-specific cultural considerations and ethical standards. The study was cross-sectional, with no longitudinal follow-up. Participant sample collection dates were obtained from the “collection_timestamp” metadata field and ranged from November 12, 2019 to May 11, 2023, after excluding non-date entries such as “not applicable.” Sequencing run dates, recorded separately in the “run_date” metadata field, ranged from April 18, 2021 to June 12, 2023. Recruitment dates were not available as a separate field from sample/data collection dates. The study included 1,976 participants (75% female; mean age: 45.5 years).

Fecal samples were collected using the Microsetta fecal collection kit^31^. Specifically, swabs from used toilet paper were placed into tubes containing 2 mL of 95% EtOH and stored at room temperature. In the US and UK, samples were shipped directly from participants to UC San Diego. In Mexico, Spain, and Japan, samples were first aggregated within the respective country before bulk shipped to UC San Diego. On receipt, samples were checked into the Microsetta system. Broken samples or samples that arrived without remaining EtOH were discarded. Only samples with complete information, including FFQ data, were used for sequencing and analysis. All measurements were obtained from distinct samples, with no repeated measurements from the same sample.

### Diet data collection and preparation

Participants in the US and UK completed the VioScreen™ food frequency questionnaire (FFQ) (Version 5, VioCare, Princeton, NJ)^32^, while participants in Mexico completed Version 5-Mex (VioCare, Princeton, NJ), which was adapted to include foods common in Mexico. Participants in Spain completed the PREDIMED FFQ, and the data were collected by the Spanish National Research Council (CSIC) at the Institute of Agrochemistry and Food Technology in Spain^33^. Participants in Japan took a digitized version of the FFQ developed for the Next Generation Multipurpose Cohort Study (JPHC-NEXT)^34^. Through these FFQs, participants reported their typical intake of a wide range of foods over the previous three months for Mexico, the US, and the UK, and the previous year for Spain and Japan, which allowed for a comprehensive assessment of dietary patterns and nutrient intake. The FFQ provided detailed estimates of macronutrient (e.g., carbohydrates, proteins, fats) and micronutrient (e.g., vitamins, minerals) intake, as well as a breakdown of food categories (e.g., fruits, vegetables, meats). To improve comparability across cohorts, dietary data were harmonized using VioScreen food codes and collapsed to common food groupings. Original food names and their corresponding grouped categories are provided in Supplemental Table 1. Any nutrient or food columns that were not measured for an entire country were excluded. After cleaning, 60 food groups and 30 nutrient variables (including calories) remained. All food and nutrient values were standardized by total calorie intake prior to analysis.

The Healthy Eating Index–2015 (HEI–2015), previously validated for use in Japan^35^, was calculated for each country using the Food Patterns Equivalents Database (FPED), following code adapted from the sample SAS scripts available on the NIH HEI website (https://github.com/lkhatib/Calculating_HEI_Python). For Japan and Spain, FPED components were derived by matching foods in each country’s dataset to their corresponding items in the Food and Nutrient Database for Dietary Studies (FNDDS; see Source Data). The matched food items were then converted to FPED component values through matrix multiplication.

Fermented food items were identified from FFQ-derived food groups within the Japanese cohort based on established definitions of microbial fermentation. Foods were included if they were consistently classified as fermented, including items such as natto, yogurt, kimchi, fermented soy products, and pickled vegetables. These categories were defined a priori based on standardized nutritional classifications.

### DNA extraction and sequencing

DNA extraction was performed using the Matrix Method as described previously by Brennan et al.^36^. Briefly, swabbed samples were transferred to Matrix Tubes^TM^. Thirty microliters of 0.1, 0.5, and 1-mm zirconia-silica beads were added to each Matrix Tube using a LabTie bead dispenser (Molgen, Netherlands). Six hundred microliters of Lysis buffer was added to each tube using the Biotek Multiflo bulk reagent dispenser (Agilent, CA, USA). The Matrix Tubes were capped using the Capit-All (ThermoFisher Scientific, MA, USA). Bead beating was performed on the SpexMiniG for 2 min at 1200 × *g*. The remaining steps of the DNA extraction followed the manufacturer’s instructions, with bead clean-ups performed using the automated KingFisher Flex Purification System (ThermoFisher Scientific, MA, USA). Extracted nucleic acids were stored at −80 °C until further processing.

DNA was prepared for shotgun metagenomics sequencing as described previously^37^. Metagenomic libraries were normalized by iSeq (Illumina) read count distribution to generate a final pool that made sequencing on the NovaSeq more efficient^38^. The iSeq-normalized pools were run on a NovaSeq 6000 (Illumina, CA, USA). The reads are available through the European Bioinformatics Institute (EBI) (accession number: PRJEB11419).

### Metagenomic data processing

After sequencing, binary base call (BCL) files were demultiplexed, converted to per-sample FASTQs, and quality-controlled and human-sequence filtered using the Qiita^39^ admin plugin: https://github.com/qiita-spots/qp-knight-lab-processing following the recommendations from Guccione et al.^40^. In summary, adapter trimming was performed using fastp^41^. Each sample was then filtered against GRCh38.p14 and T2T-CHM13v2.0^42^ via alignment using minimap2^43^. Reads aligning to either human reference genome were discarded. Next, a pangenome index was created using the 94 reference assemblies from the Human Pangenome Reference Consotrium release 1^44^, and reads with high pseudo-matching lengths to the pangenome were discarded. The resulting per-sample-FASTQs were uploaded into Qiita study ID #10317 (https://qiita.ucsd.edu/study/description/10317; Prep IDs: 16766, 16762, 16761, 16773, 16780, 16756, 16842, 16848, 16849, 16853, 16841, 16854, 16875) and processed with its default processing workflow. The metagenomic workflow maps paired-end reads using Bowtie2 v2.5.4^45^ against Web of Life release 2 (WoL2)^46^ with the SHOGUN parameter set^47^. A feature table is then constructed using woltka v0.1.7^48^.

The listed Prep IDs were aggregated into a single analysis on Qiita^39^. Coverage values were calculated using MIcrobiome COVerage (micov)^49^, and OGUs with <1% cumulative coverage were excluded.

Quality control steps included removing blank and non-THDMI samples. Samples with invalid entries in any of the following metadata fields were excluded: thdmi_cohort, host_age, sex, antibiotic_history, covid_level_of_wellbeing, and host_body_mass_index. We further removed samples missing dietary information. An additional 58 samples from Mexico were excluded due to technical bias introduced by re-sequencing following low read quality. Finally, samples with feature sums less than or equal to 1,000,000 reads were filtered out.

After these quality control procedures, the resulting feature table contained 1976 samples (Japan: 410; Mexico: 397; Spain: 450; UK: 308; US: 411) and 3375 microbial OGUs.

### Metadata data processing

All metadata variables, including sex and age, were self-reported. Metadata were subjected to quality control and harmonization. Age values ≤18 or ≥100 were set to NaN and subsequently removed, while body mass index (BMI) values <12 or >70 were also set to NaN and excluded, consistent with previous work^9^. Categorical variables with fewer than two unique values within any country were dropped, as were binary columns with fewer than 10 individuals in one group per country.

Several variables were collapsed into broader categories to reduce sparsity and ensure sufficient sample sizes within each group across countries, enabling robust cross-country comparisons. Specifically, plant diversity was reduced to “Less than 10” and “More than 30”; and antibiotic history was reduced to “No use in the past year” and “Within the last month”, as previously described^7^. Clinical diagnosis columns were collapsed to “Diagnosed by a medical professional (doctor, physician assistant)” and “I do not have this condition”^50^. A binary “healthy” versus “unhealthy” variable was created following prior methodology^51^. Columns describing contraceptive and deodorant use were reduced to True/False. Home-cooked meals frequency was collapsed to “Daily” vs. “Not daily”, and drinks per session were simplified to “Less than 2” vs. “More than 2”.

Finally, columns with fewer than five samples in any group within a country were removed from the analysis.

### MAG assembly, binning, and annotation

MaxBin 2 v2.27^52^, MetaBAT 2 v2.18^53^, and CONCOCT v1.10^54^ were individually applied for contig binning with default parameters. The resulting Metagenome-Assembled Genomes (MAGs) were first refined (quality improvement) using DAS_Tool^55^ with the search engine USEARCH^56^. CheckM^57^ lineage_wf was then used for MAG quality assessment with default parameters. The quality score was defined as completeness − 5*contamination + Strain heterogeneity*0.5. Medium to high quality MAGs were defined as quality scores larger than 0.5. Dereplication of the MAGs was performed using dRep (v2.2.4)^58^ with an ANI threshold of 95% by the -fastANI option (minimum completeness 70%, maximum contamination 10%, and quality score > 0.5 for filtering MAGs before dereplication). Quality information from CheckM was passed to dRep to select the best-quality MAG as representative of each resulting 95% ΑΝΙ cluster. MAGs were classified against the Genome Taxonomy Database v214^59^ using the GTDB-Tk v2.4.3 classify_wf workflow^60^.

### Microbiome data representations

To capture microbiome variation across multiple biological scales, we analyzed data at the level of Operational Genomic Units (OGUs; reference genome-aligned features) and genome-resolved units (MAGs-derived species bins) (Fig. S1).

### Operational genomic units (OGUs)

OGUs were defined as reference genome-aligned features, representing individual reference genomes from the WoL2 curated database. Shotgun metagenomic reads were aligned directly to reference genomes, and each genome was treated as a distinct feature without aggregation into taxonomic groups. This approach preserves maximal resolution and avoids information loss associated with taxonomic binning. Thus, multiple OGUs may correspond to closely related strains within a species (e.g., multiple *Escherichia coli* reference genomes).

### Metagenome-assembled genomes (MAGs)

MAGs were reconstructed through assembly and binning of metagenomic reads. High-quality MAGs were retained based on completeness and contamination thresholds and dereplicated to define species-level genome bins as described above.

### Species-level bins

Species-level bins represent clusters of MAGs grouped based on genomic similarity (e.g., ≥95% average nucleotide identity), approximating species-level groupings. These were used for genome-resolved and strain-level analyses.

Unless otherwise specified, multivariate analyses were performed using OGU-level profiles (Figs. 1–3), while genome-resolved analyses were used to investigate strain- and gene-level variation (Fig. 4).

### Statistical analysis

#### Demographic, nutrient, and food summaries

Demographic characteristics, nutrient intake, and food group distributions were summarized across countries. Kruskal–Wallis tests were used for continuous variables and Chi-squared tests for categorical variables.

### Principal component analyses (PCA)

Robust Principal Component Analysis (RPCA)^61^, implemented in gemelli v0.0.9 (https://github.com/biocore/gemelli), was used to analyze microbiome composition to provide PCA-like embeddings comparable to those used for other data layers. For metadata, diet-related and COVID-19–related variables were excluded. Ordinal variables were encoded using OrdinalEncoder (scikit-learn v0.24.1), and non-ordinal categorical variables were one-hot encoded. Missing values were imputed using SimpleImputer with the mean strategy. Metadata, nutrient, and food data were standardized with StandardScaler. PCA was performed using sklearn.decomposition. Pairwise distance matrices were computed, and PERMANOVA tests were conducted using scikit-bio v0.5.7^62^. To assess whether age, sex, cosmetics use, BMI, and sequencing depth confounded geographic clustering in microbiome data, we evaluated associations between country and metagenomic profiles using PERMANOVA (adonis2^63^ in R), including these variables as covariates. Sensitivity analyses were additionally performed in a female-only subset and in a subset of individuals with BMI in the normal range.

### Sparse canonical correlation analysis (sCCA)

Sparse canonical correlation analysis was conducted between metadata, foods, nutrients, and metagenomic data. Encoded and imputed metadata, and nutrient and food inputs from the PCA preprocessing described above were used. Metagenomic data were preprocessed with matrix_rclr and MatrixCompletion (gemelli.preprocessing). Sparse CCA was performed using SCCA_IPLS from cca-zoo v2.6.0^18^. Data were standardized within the function, and columns with zero variance were excluded. Tuning parameters (α and L1 ratio) were selected using 5-fold cross-validation and the one–standard error (1-SE) rule.

### Prevalence analysis

For each country, the prevalence of each microbial taxon was calculated as the proportion of samples in which the taxon was detected ( > 0 counts). Ubiquitous OGUs were defined as having >99% prevalence across all countries and excluded from comparative analyses. We first searched for “exclusive” OGUs, defined as those with ≥80% prevalence in one country and 0% in all others, and no OGUs met this criterion. We then lowered the threshold to identify OGUs with ≥60% prevalence in one country and ≤10% prevalence in all others. Enriched OGUs were defined as having a prevalence ratio ≥3 relative to the next-highest country.

Differential abundance was assessed using Bayesian Inferential Regression for Differential Microbiome Analysis (BIRDMAn)^21^ (v0.1.0), testing metadata, nutrient, and food variables with consistent variance across countries. Variables to test were selected based on those that showed the greatest variation across countries (Fig. 1). All associations were adjusted for thdmi_cohort. Metadata variables tested included: host_age, bowel_movement_quality_renamed, unhealthy, host_body_mass_index, sex, cosmetics_frequency, vitamin_d_supplement_frequency, vitamin_b_supplement_frequency, multivitamin, autoimmune_reduced, probiotic_frequency, thyroid_reduced, drinks_per_session_reduced. Nutrients tested included: isoflavones_total, iron, fiber, fiber_water, protein, carbohydrates, monounsaturated fat, polyunsaturated fat, cholesterol, vitamin B6, potassium, magnesium, thiamin, niacin_equivalents, zinc, folate_total, vitamin C, insoluble fiber, and total fat. Foods tested included: garlic, rice/pasta/noodles, yogurt, processed meats, salt, soups/creams.

The WoL2 reference phylogeny was pruned to include only the enriched OGUs and uploaded to Interactive Tree Of Life (iTOL) version 7^64^. In iTOL, branch-length scaling was disabled to emphasize taxonomic topology rather than evolutionary distance. BIRDMAn association results were encoded as upward or downward arrows to indicate the direction of association and were added as annotation tracks in iTOL.

### HEI across countries

Healthy Eating Index (HEI) total scores were compared across countries using analysis of variance (ANOVA) from scipy.stats v1.10.1^65^. Associations between HEI scores and metagenomic profiles were assessed using the adonis2^63^ function in R, adjusting for sex, age, and BMI.

To test whether microbiome OGUs associated with HEI in one country could generalize to other countries (US, Mexico, Spain), we applied an Elastic Net regression framework for both within- and cross-country prediction. Prior work has shown that using presence–absence representations for Elastic Net regression performs as well as, or better than, abundance-based transformations for predictive tasks^66^. Therefore, metagenomic OGUs were converted to presence–absence. To remove noisy or redundant features, OGUs were retained only if they were present in at least 5% of samples. To prioritize OGUs associated with dietary quality, we conducted a supervised univariate screen using Pearson correlations between each taxon and Healthy Eating Index (HEI) scores, followed by false discovery rate (FDR) correction (*q* ≤ 0.25). To address redundancy, we applied correlation pruning to remove OGUs with pairwise |*ρ*| ≥ 0.95, keeping one representative per correlated group.

We trained Elastic Net regression models (sklearn.linear_model.ElasticNetCV) using 5-fold cross-validation to optimize model hyperparameters, including the L1 ratio and regularization strength (*α*). Prior to fitting, OGUs were standardized to unit variance. Models were first evaluated within countries (e.g., 5-fold cross-validation within the Mexico, US, and Spain cohorts). To assess cross-cohort generalization, we additionally trained models on one country and evaluated predictive performance in another (e.g., train on Mexico, test on US). The same feature selection and filtering steps were applied based solely on the training cohort, and the resulting feature set was applied to the test cohort (with missing OGUs set to zero).

BIRDMAn models were then run separately within each country with HEI total scores as the outcome. For each country, the log ratio of positively versus negatively associated OGUs was computed, and Spearman correlations were calculated between these log ratios and the total HEI score within each country. Upset plots were generated to compare the number of OGUs associated with HEI across countries.

The WoL2 reference phylogeny was pruned to include the OGUs that were significantly correlated with HEI in all five countries and uploaded to iTOL^64^. Spearman correlations were computed between CLR-transformed abundances and HEI total scores. Mean CLR-transformed values and prevalence for each country were then calculated and visualized on the phylogenetic tree.

### Country-specific genomic differentiation of microbial species

We assembled and quality-filtered metagenome-assembled genomes (MAGs) at ≥80% completeness and ≤10% contamination. MAGs were assigned to species-level bins using an ANI-based map, and all downstream analyses were performed within each species bin.

To ensure sufficient statistical power for population-level comparisons, analyses were restricted to species with at least 30 high-quality MAGs across cohorts. This threshold was selected to provide adequate sample size per country for downstream statistical testing (e.g., PERMANOVA and genome-wide association analyses). To assess biological prevalence independently of MAG recovery, quality-controlled shotgun metagenomic reads were mapped back to the dereplicated MAG database using the same read-mapping workflow and software versions described above for metagenomic profiling. Coverage statistics were calculated using micov. For each species-level genome bin retained in the genome-resolved analysis, we summarized MAG recovery, breadth-based prevalence, and mapped-read abundance across cohorts. MAG recovery was reported as the number of recovered MAGs per 100 samples. Species prevalence was defined as the percentage of samples within each cohort with ≥40% genome breadth at ≥10× read depth, using the ≥40% genome-breadth threshold as a conservative cutoff for species detection. Abundance was summarized as the median percentage of mapped reads within each cohort. This read-mapping analysis was used as a complementary assessment of species detection and abundance and was not used to define the ≥30 MAG threshold for genome-resolved PERMANOVA or GWAS analyses.

Panpiper-kit was used to compute associations between genomic distances within a species and country of origin. For each pair of MAGs within a species bin, we computed pairwise Mash^23^ distances (1-ANI) using *k* = 18 and *s* = 5000 (p), which sketch genomes and provide a fast, MinHash-based approximation to overall sequence divergence. Using each species’ Mash distance matrix, we tested whether MAG distances were associated with country. We used PERMANOVA (9999 permutations) for the categorical factor (“country”) and report the test statistic and permutation p-value.

For species where a country level had ≥30 genomes, we performed unitig-based GWAS with pyseer^24^ v1.4.0, using unitigs generated by unitig-caller. Unitigs capture fine-scale signals across the pangenome, including accessory genes and intergenic/regulatory variants. We ran binary contrasts between a focal country vs. all others and controlled multiple testing with Benjamini–Hochberg FDR. Significant unitigs were collapsed by overlapping coordinates and mapped to genes on a representative genome; genes were annotated with Bakta^67^, and results were summarized by gene and pathway for interpretation.

To investigate finer-resolution patterns, we selected two species (*Bifidobacterium longum* and *Faecalibacterium longum*) with high MAG recovery across all five countries and significant PERMANOVA results for gene presence/absence analysis. For these specific species of interest, we reduced the feature space to gene presence/absence (PAV) to maintain power with small sample sizes. For each gene, we tested presence in the focal country vs. others (2 × 2 table) and controlled FDR across genes. This yields interpretable pathway-level signals with a far lower multiple-testing burden than unitig GWAS.

All distance- and GWAS-level steps were orchestrated with panpiper-kit (ppk-run CLI). Key settings were: --perms 999, --mash-k 18, --mash-s 5000, and --kmer 31. The toolkit writes lineage-level association tables (PERMANOVA results) and per-species pyseer outputs (BH-adjusted) as plain TSVs for downstream statistics and figures.

All statistical tests were two-sided unless otherwise specified. For Bayesian and machine learning approaches, no directional priors or one-sided hypotheses were imposed. Where applicable, exact *P* values are reported; otherwise, thresholds are provided for very small values.

### Reporting summary

Further information on research design is available in the Nature Portfolio Reporting Summary linked to this article.

## Supplementary information

Supplementary Information

Reporting Summary

Transparent Peer Review File

## Source data

Source Data

## Data Availability

Raw shotgun metagenomic reads, per-sample FASTQ files, processed tables, and metadata generated or analyzed in this study are available through the following repositories: European Bioinformatics Institute (EBI): PRJEB11419 (raw shotgun metagenomic sequencing reads deposited in the European Nucleotide Archive/European Bioinformatics Institute). Qiita: 10317 (Qiita study containing per-sample FASTQ files, processed tables, and metadata; Prep IDs: 16766, 16762, 16761, 16773, 16780, 16756, 16842, 16848, 16849, 16853, 16841, 16854, 16875). Dietary intake data used in this study are provided as Source Data. Source data are provided with this paper.

## Code availability

Data processing and statistical analysis scripts are available at: https://doi.org/10.5281/zenodo.21609747.
