# Supplementary material for: Five-country analysis of geographic and dietary drivers of gut microbiome composition and genomic variation
Source: Nat Commun. 2026 Sep 21;17:11004. doi: 10.1038/s41467-026-77928-9 (PMC13687547; doi:10.1038/s41467-026-77928-9)
Supplement: Supplementary file 1 [file 41467_2026_77928_MOESM1_ESM.pdf]

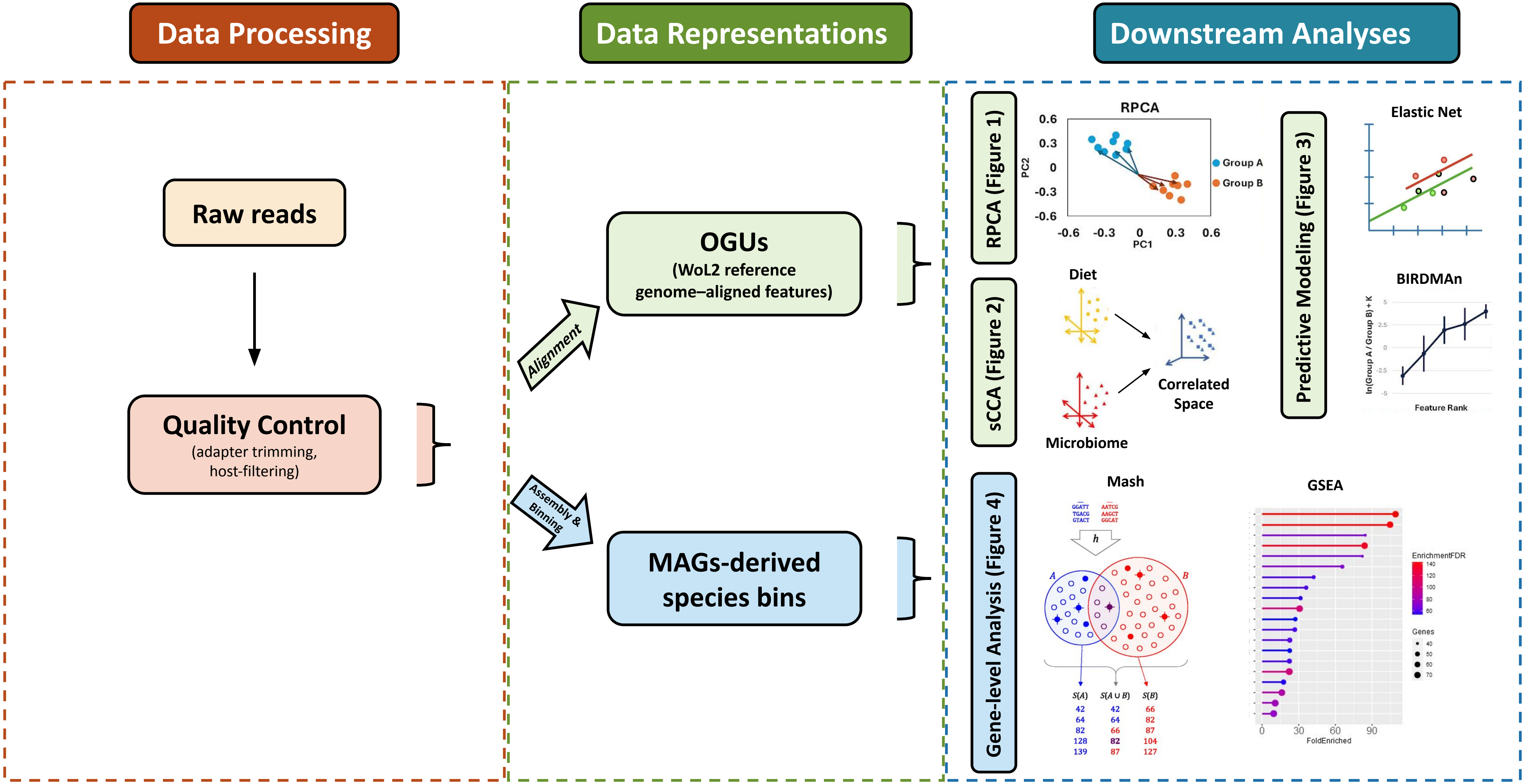

Figure S1. Overview of microbiome data processing, representations, and downstream analyses. Shotgun metagenomic sequencing reads were subjected to quality control, including adapter trimming and host read removal. Processed reads were then analyzed along two complementary paths. In the alignment-based approach, reads were mapped to the WoL2 reference genome database to generate Operational Genomic Units (OGUs), which represent individual reference genomes and enable limited strain-level resolution without taxonomic aggregation. In parallel, reads were assembled and binned to generate metagenome-assembled genomes (MAGs), which were subsequently clustered into species-level bins for genome-resolved analyses. These data representations were used for downstream analyses across multiple biological scales. OGU-based profiles were used for multivariate analyses, including robust principal component analysis (RPCA; Fig. 1) and sparse canonical correlation analysis (sCCA; Fig. 2), as well as predictive modeling approaches (Fig. 3), including elastic net regression and Bayesian regression (BIRDMAn). Genome-resolved species bins were used for gene-level analyses (Fig. 4), including gene presence/absence comparisons and functional enrichment analyses (e.g., Mash-based comparisons and gene set enrichment analysis, GSEA). This framework enables integration of microbiome composition, strain-level variation, and functional potential in relation to dietary and geographic factors.

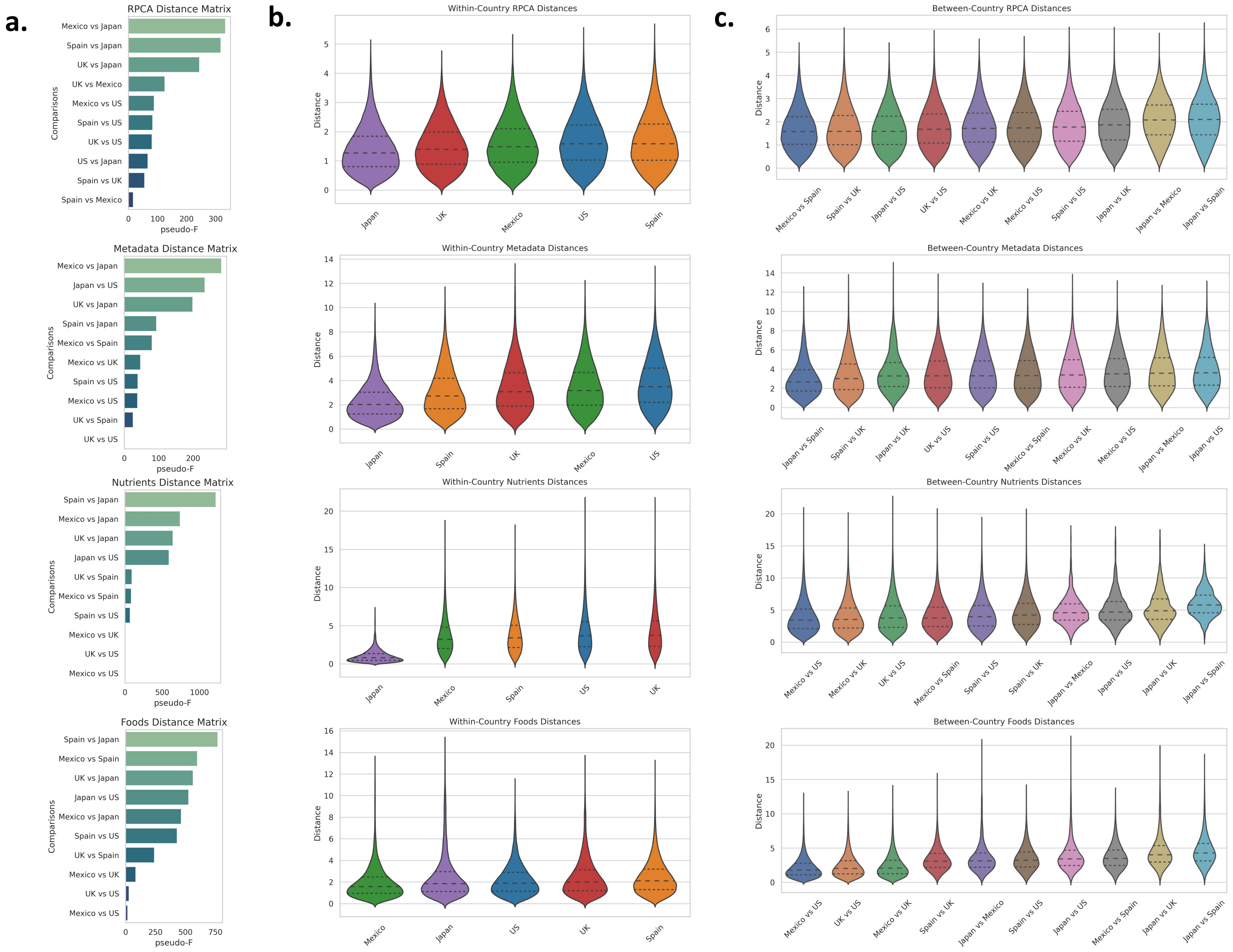

Fig S2. Cross-country differences in microbiome composition, metadata, nutrients, and foods.
(a) Pairwise PERMANOVA pseudo-F statistics comparing country-level dissimilarities across four data types: microbiome, metadata, nutrients, and foods. Higher pseudo-F values reflect greater between-country separation (n = 1,976). RPCA = Robust Principal Component Analysis. (b) Within-country distributions of pairwise distances for microbiome (top), metadata (middle), nutrients (third), and foods (bottom). Violin plots illustrate the distribution density of within-country variation, with dashed lines indicating quartiles. (c) Between-country pairwise distances for microbiome (top), metadata (middle), nutrients (third), and foods (bottom). Each violin represents distances between two countries (e.g., Mexico–Spain, Spain–Japan).

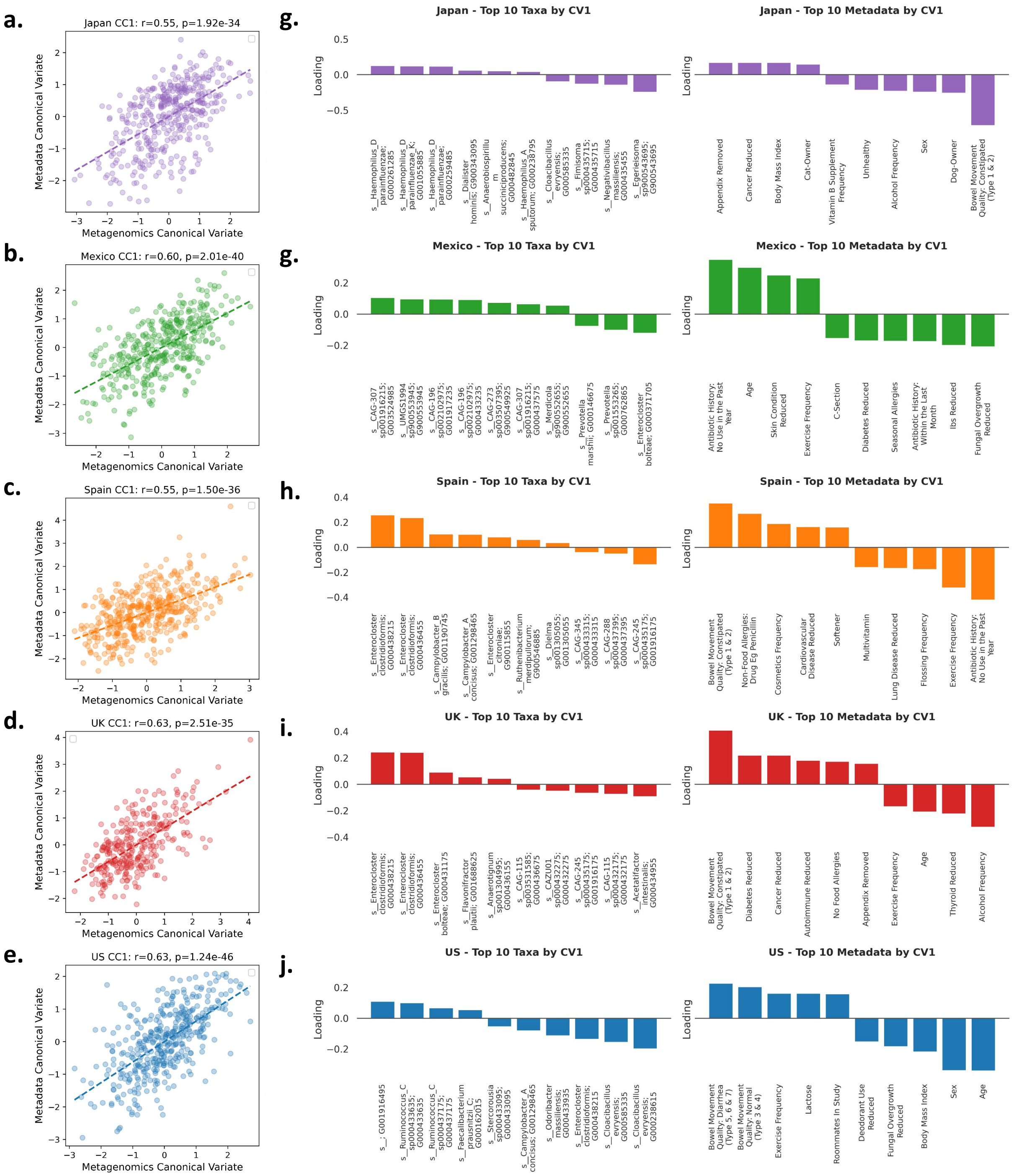

Figure S3. Sparse canonical correlation analysis (sCCA) of metagenomics with metadata within countries. (a–e) Scatterplots of canonical variates (CVs) from sCCA showing correlations between metagenomics and metadata within (a) Japan (n = 410), (b) Mexico (n = 397), (c) Spain (n = 450), (d) UK (n = 308), and (e) US (n = 411). Each point represents an individual with best-fit regression lines for each cohort. Pearson correlation coefficients (r) and p-values are shown for each country. CC1 = Canonical Correlation 1. (f-j) Barplots of the top 10 features with the largest absolute loadings on canonical variate 1 (CV1) for microbial taxa (left) and metadata (right) within (f) Japan, (g) Mexico, (h) Spain, (i) UK, (j) US. Positive and negative bars indicate direction of association.

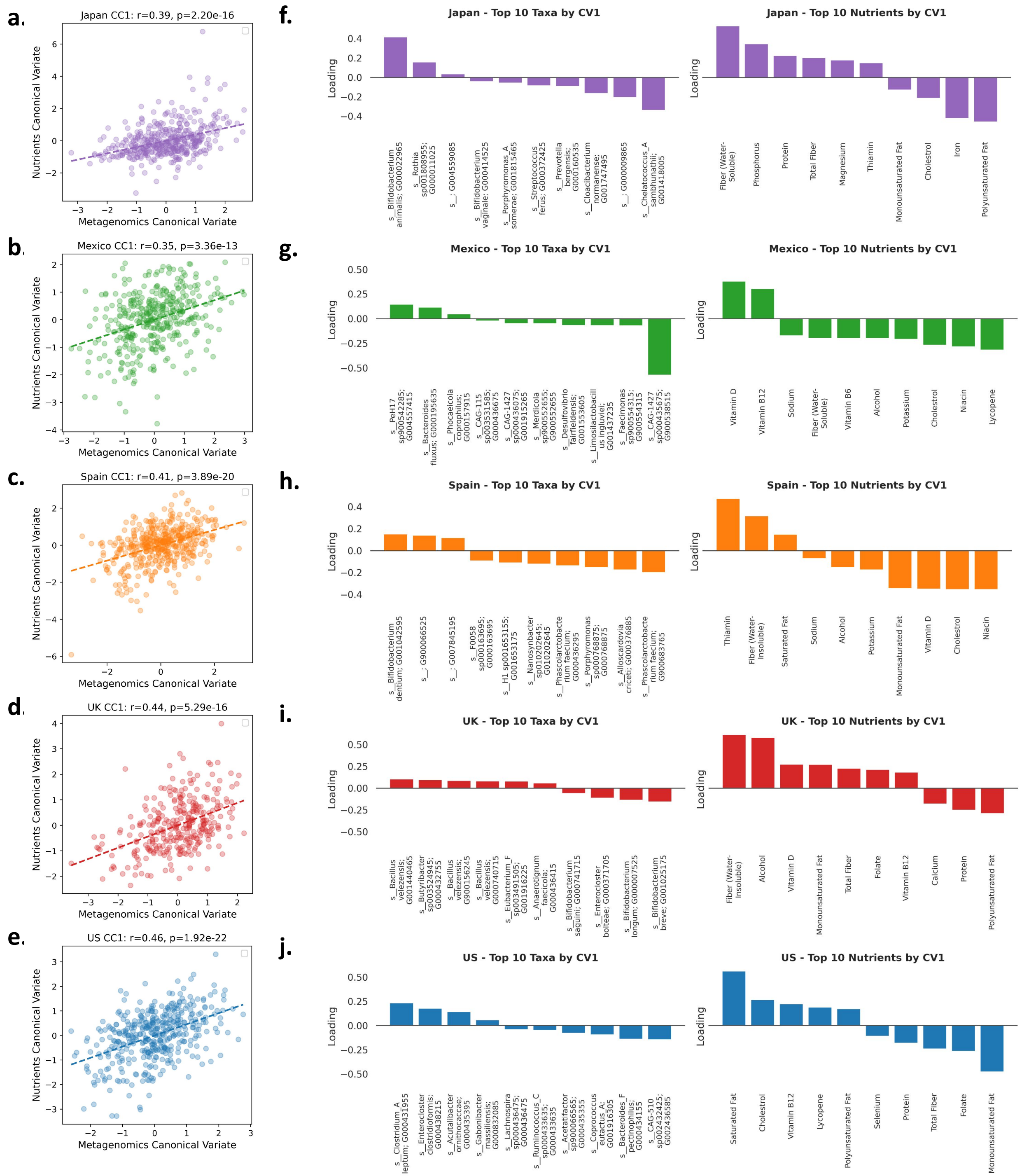

Figure S4. Sparse canonical correlation analysis (sCCA) of metagenomics with nutrients within countries. (a–e) Scatterplots of canonical variates (CVs) from sCCA showing correlations between metagenomics and nutrients within (a) Japan (n = 410), (b) Mexico (n = 397), (c) Spain (n = 450), (d) UK (n = 308), and (e) US (n = 411). Each point represents an individual with best-fit regression lines for each cohort. Pearson correlation coefficients (r) and p-values are shown for each country. CC1 = Canonical Correlation 1. (f-j) Barplots of the top 10 features with the largest absolute loadings on canonical variate 1 (CV1) for microbial taxa (left) and nutrients (right) within (f) Japan, (g) Mexico, (h) Spain, (i) UK, (j) US. Positive and negative bars indicate direction of association.

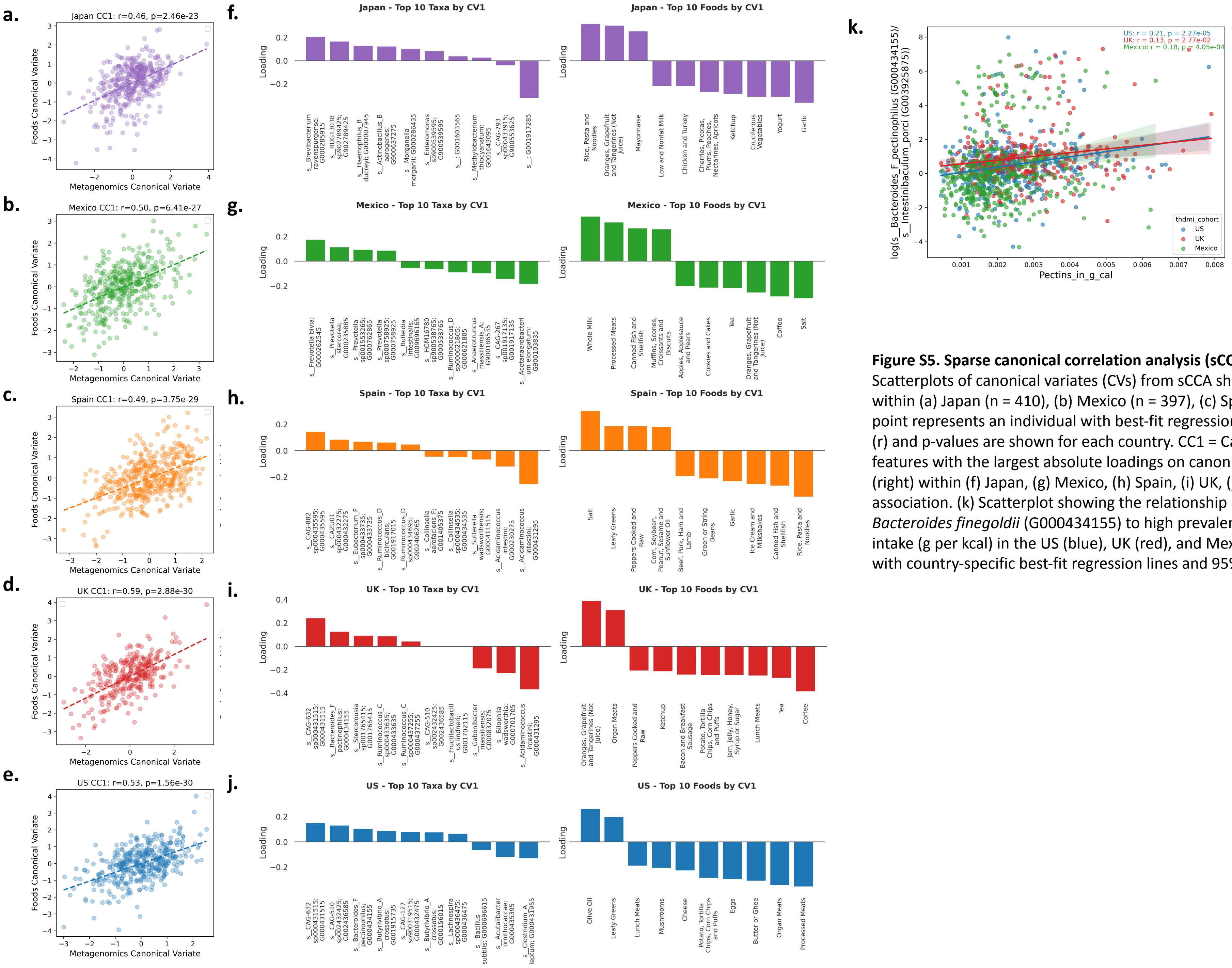

Figure S5. Sparse canonical correlation analysis (sCCA) of metagenomics with foods within countries. (a–e) Scatterplots of canonical variates (CVs) from sCCA showing correlations between metagenomics and foods within (a) Japan (n = 410), (b) Mexico (n = 397), (c) Spain (n = 450), (d) UK (n = 308), and (e) US (n = 411). Each point represents an individual with best-fit regression lines for each cohort. Pearson correlation coefficients (r) and p-values are shown for each country. CC1 = Canonical Correlation 1. (f-j) Barplots of the top 10 features with the largest absolute loadings on canonical variate 1 (CV1) for microbial taxa (left) and foods (right) within (f) Japan, (g) Mexico, (h) Spain, (i) UK, (j) US. Positive and negative bars indicate direction of association. (k) Scatterplot showing the relationship between the log-transformed abundance ratio of *Bacteroides finegoldii* (G000434155) to high prevalence taxa *Intestinibaculum porci* (G003925875) and pectin intake (g per kcal) in the US (blue), UK (red), and Mexico (green). Points represent individual participants, with country-specific best-fit regression lines and 95% confidence intervals overlaid.

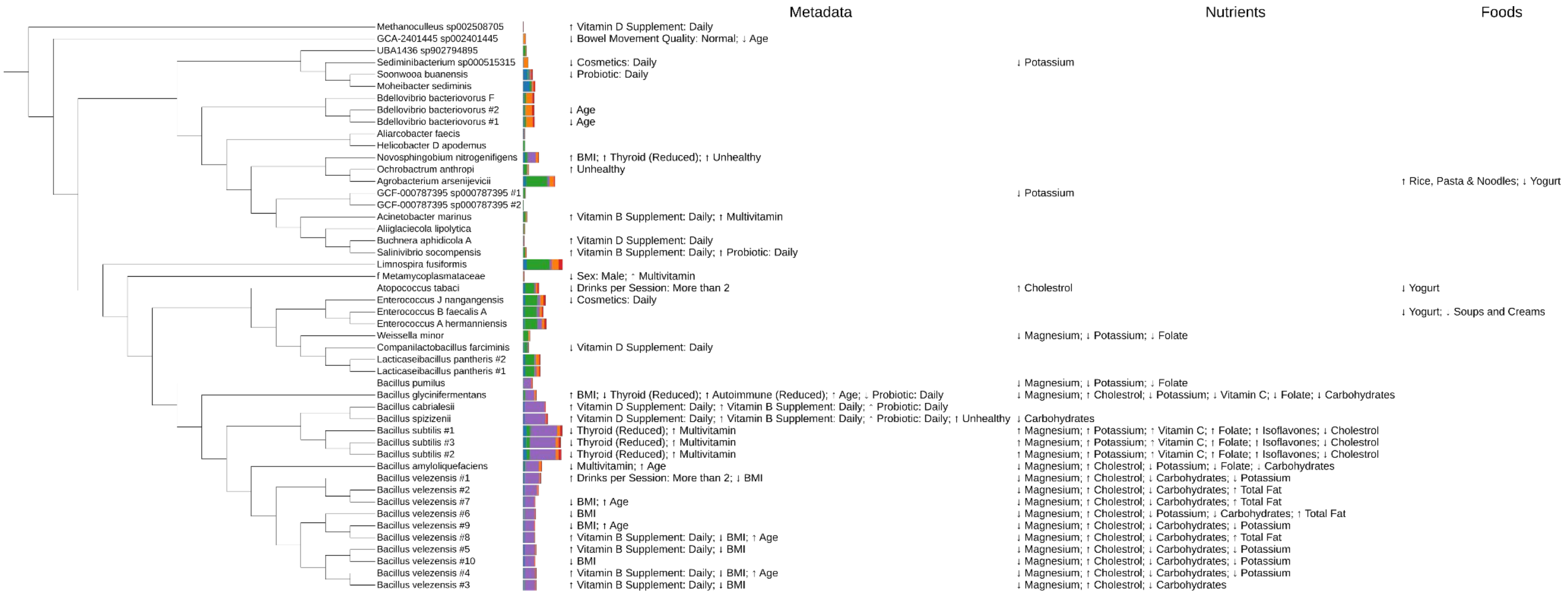

Figure S6. Phylogenetic tree of enriched microbial taxa. The phylogenetic tree depicts country-enriched microbial taxa presenting significant associations with metadata, nutrients or foods, identified using BIRDMAn. Bar plots at the tree tips show the prevalence of each taxon by country (purple = Japan (n = 410), green = Mexico (n = 397), orange = Spain (n = 450), red = UK (n = 308), blue = US (n = 411)). Text labels indicate associations with metadata (left), nutrients (middle), and foods (right). Upward arrows denote positive associations, while downward arrows denote negative associations. All associations were adjusted for thdmi_cohort. Branch lengths were disabled during visualization to emphasize taxon relationships rather than evolutionary distances.

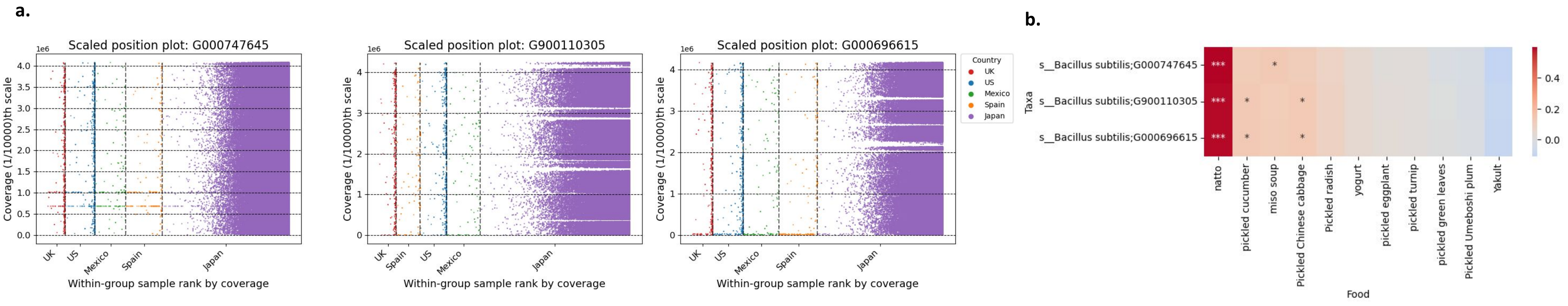

Figure S7. Coverage of *B. subtilis* OGUs and associations with fermented foods. (a) Scaled position plots (1/10,000th genome scale) showing per-sample genomic coverage for the OGUs G000747645, G900110305, and G000696615 stratified by country (n = 1,976). Each dot represents a covered genomic bin for a sample, plotted along the x-axis by within-group sample rank (low to high coverage). Vertical dashed lines demarcate cohort boundaries. The y-axis represents genomic position bins with detected coverage. (b) Heatmap showing Spearman correlations between the CLR-abundance of three *B. subtilis* OGUs (rows) and consumption of fermented and pickled foods (columns) in participants from Japan (n = 410). Warmer colors indicate positive correlations, and cooler colors indicate negative correlations. Statistical significance is denoted by asterisks based on FDR-adjusted p-values (*p < 0.05, **p < 0.01, ***p < 0.001).

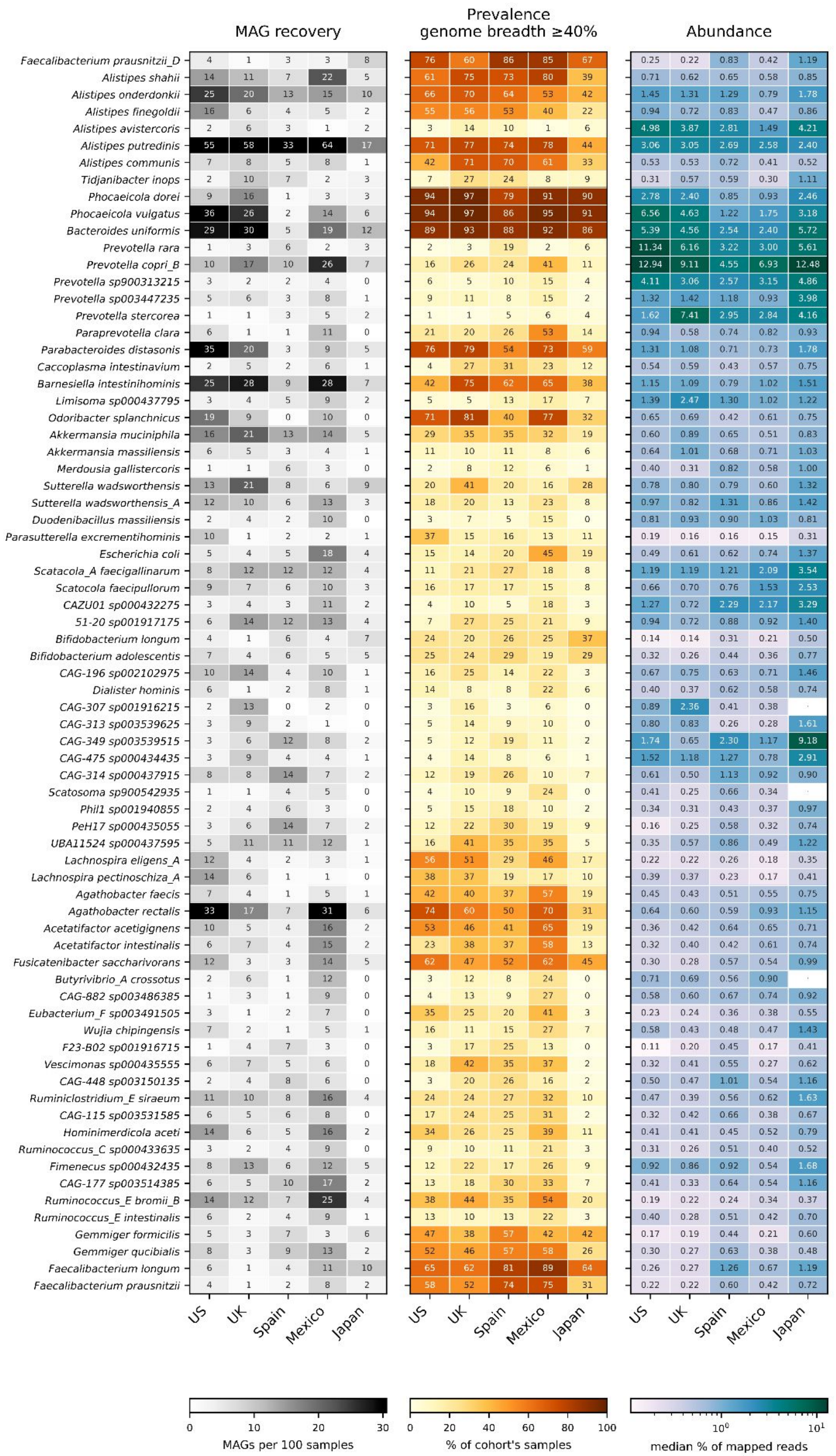

Figure S8. Cohort-stratified MAG recovery, breadth-based prevalence, and abundance for species retained in genome-resolved analyses. For each of the 73 species-level genome bins retained for genome-resolved analyses, heatmaps show MAG recovery, breadth-based prevalence, and abundance across the US (n = 411), UK (n = 308), Spain (n = 450), Mexico (n = 397), and Japan (n = 410) cohorts. MAG recovery is shown as the number of recovered MAGs per 100 samples. Prevalence was estimated by mapping metagenomic reads back to the dereplicated MAG database and calculating the percentage of samples within each cohort with ≥40% genome breadth at ≥10× read depth. Abundance is shown as the median percentage of mapped reads within each cohort. This analysis provides complementary biological context for interpreting genome-resolved differentiation because MAG recovery can be influenced by assembly success, sequencing depth, and genome characteristics.

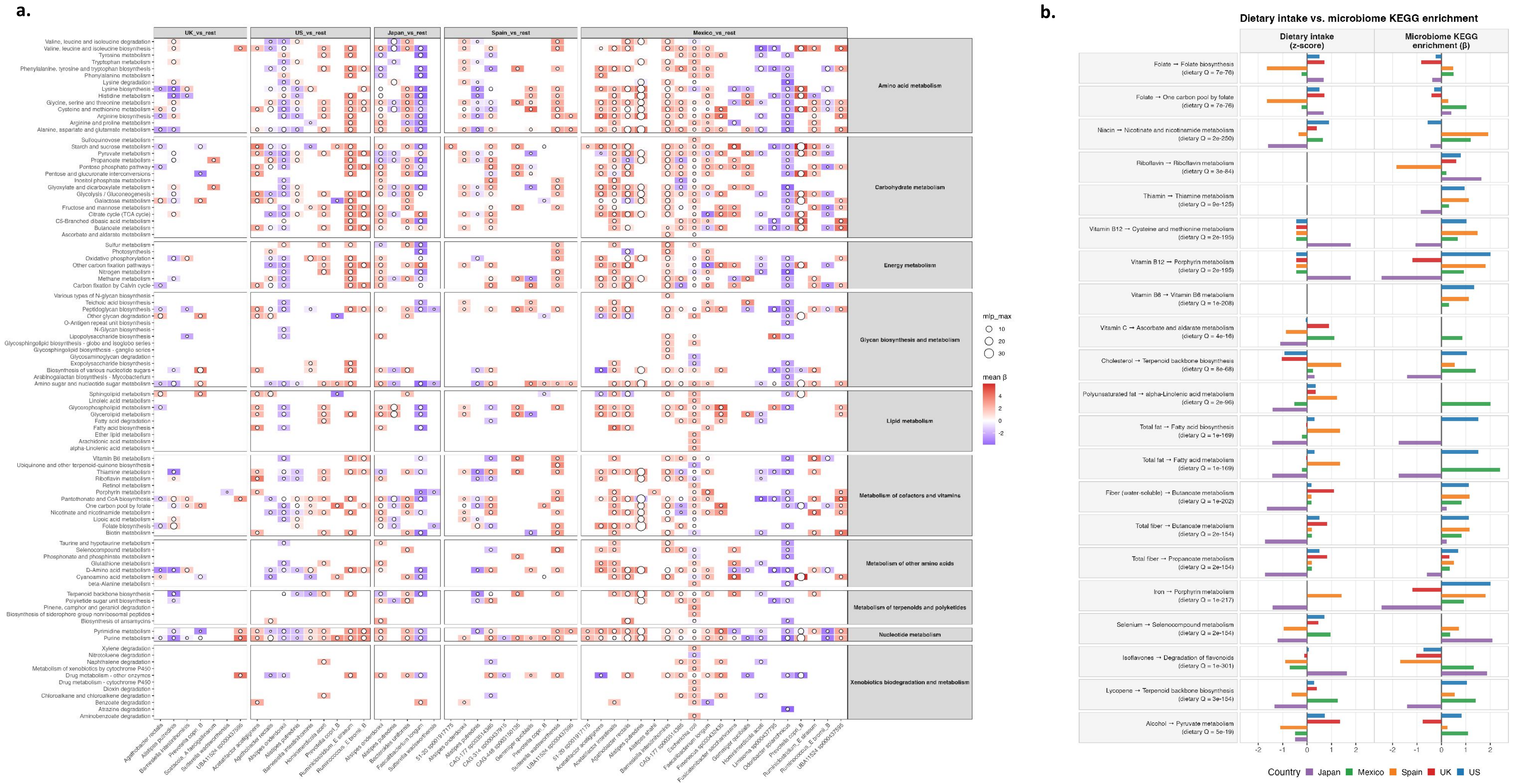

Figure S9. Cross-population enrichment of functional genes across bacterial species. (a) Heatmap of species by country comparison showing the mean effect size and magnitude of the association between a given bacterial KEGG group and origin country (n = 1,976). Tile color encodes the average effect size (mean β) from a genome-wide association study, where positive values (red) indicate enrichment in that country and negative values (blue) indicate depletion relative to the remaining cohorts. Circle overlays mark the statistical signal strength: circle size corresponds to the maximum $-\log_{10}$ p value (mlp_max) observed for that KEGG group in the respective species. Only gene–species pairs with ≥ 1 significant variant (FDR < 0.05) in at least one population are shown. (b) Left panels show standardized dietary intake (z-scored), and right panels show corresponding microbiome KEGG pathway enrichment (β coefficients). Colors indicate country. Comparisons between all nutrient intake and microbiome functional enrichment across countries (n = 1,976).

Parameter	Japan	Mexico	Spain	UK	US	Statistic	Q-value
Vitamin C	0.068 ± 0.013	0.098 ± 0.066	0.071 ± 0.029	0.095 ± 0.051	0.082 ± 0.049	78.419	3.77E-16
Alcohol	0.003 ± 0.005	0.003 ± 0.005	0.002 ± 0.002	0.006 ± 0.008	0.005 ± 0.007	91.891	5.40E-19
Calories	1757.161 ± 166.074	2289.243 ± 898.876	2075.074 ± 796.898	2011.354 ± 690.252	2018.798 ± 678.109	100.87	6.88E-21
Vitamin D	0.005 ± 0.001	0.004 ± 0.002	0.005 ± 0.003	0.004 ± 0.002	0.003 ± 0.002	275.461	2.36E-58
Protein	0.040 ± 0.001	0.042 ± 0.008	0.049 ± 0.011	0.038 ± 0.008	0.040 ± 0.009	318.149	1.52E-67
Cholestrol	0.165 ± 0.010	0.163 ± 0.081	0.195 ± 0.118	0.128 ± 0.087	0.131 ± 0.095	319.537	7.92E-68
Potassium	1.581 ± 0.160	1.696 ± 0.471	2.015 ± 0.402	1.898 ± 0.457	1.666 ± 0.405	351.861	8.69E-75
Folate	0.223 ± 0.030	0.202 ± 0.057	0.169 ± 0.049	0.224 ± 0.067	0.219 ± 0.064	356.91	7.37E-76
Saturated Fat	0.010 ± 0.001	0.012 ± 0.003	0.014 ± 0.003	0.013 ± 0.005	0.013 ± 0.004	390.409	4.48E-83
Riboflavin	0.001 ± 0.000	0.001 ± 0.000	0.001 ± 0.000	0.001 ± 0.000	0.001 ± 0.000	395.794	3.22E-84
Phosphorus	0.609 ± 0.042	0.763 ± 0.121	0.676 ± 0.117	0.697 ± 0.115	0.693 ± 0.125	426.166	9.25E-91
Polyunsaturated Fat	0.007 ± 0.000	0.008 ± 0.002	0.010 ± 0.003	0.009 ± 0.002	0.009 ± 0.002	452.375	2.10E-96
Zinc	0.005 ± 0.000	0.006 ± 0.001	0.006 ± 0.001	0.005 ± 0.001	0.006 ± 0.001	498.137	2.82E-106
Thiamin	0.001 ± 0.000	0.001 ± 0.000	0.001 ± 0.000	0.001 ± 0.000	0.001 ± 0.000	583.686	9.28E-125
Fiber (Water-Insoluble)	0.006 ± 0.001	0.010 ± 0.003	0.010 ± 0.004	0.011 ± 0.004	0.011 ± 0.004	621.725	5.77E-133
Calcium	0.334 ± 0.052	0.557 ± 0.158	0.492 ± 0.150	0.565 ± 0.165	0.545 ± 0.195	683.741	2.31E-146
Lycopene	1.033 ± 0.255	3.586 ± 1.832	1.712 ± 1.223	2.736 ± 1.716	2.634 ± 1.836	720.076	3.36E-154
Selenium	0.042 ± 0.001	0.059 ± 0.012	0.044 ± 0.012	0.055 ± 0.012	0.057 ± 0.013	720.917	2.38E-154
Total Fiber	0.008 ± 0.001	0.014 ± 0.005	0.014 ± 0.005	0.016 ± 0.006	0.015 ± 0.005	721.97	1.52E-154
Magnesium	0.164 ± 0.014	0.190 ± 0.046	0.267 ± 0.067	0.208 ± 0.046	0.198 ± 0.045	740.732	1.44E-158
Total Fat	0.032 ± 0.002	0.039 ± 0.007	0.048 ± 0.009	0.040 ± 0.010	0.042 ± 0.009	791.702	1.44E-169
Vitamin B12	0.004 ± 0.000	0.002 ± 0.001	0.002 ± 0.001	0.002 ± 0.001	0.002 ± 0.001	911.163	2.12E-195
Sodium	2.160 ± 0.083	1.881 ± 0.499	1.295 ± 0.368	1.818 ± 0.581	1.914 ± 0.602	911.19	2.12E-195
Fiber (Water-Soluble)	0.002 ± 0.000	0.004 ± 0.001	0.004 ± 0.001	0.005 ± 0.002	0.004 ± 0.002	945.569	9.55E-203
Carbohydrates	0.143 ± 0.004	0.122 ± 0.020	0.090 ± 0.022	0.119 ± 0.026	0.114 ± 0.025	953.314	2.34E-204
Vitamin B6	0.001 ± 0.000	0.001 ± 0.000	0.001 ± 0.000	0.001 ± 0.000	0.001 ± 0.000	973.57	1.14E-208
Monounsaturated Fat	0.012 ± 0.000	0.014 ± 0.004	0.024 ± 0.006	0.015 ± 0.005	0.016 ± 0.005	984.099	7.47E-211
Iron	0.005 ± 0.001	0.007 ± 0.001	0.009 ± 0.002	0.007 ± 0.002	0.007 ± 0.002	1016.046	1.19E-217
Niacin	0.010 ± 0.001	0.019 ± 0.004	0.015 ± 0.003	0.018 ± 0.003	0.020 ± 0.005	1167.736	2.36E-250
Isoflavones	0.013 ± 0.009	0.001 ± 0.002	0.000 ± 0.000	0.004 ± 0.006	0.005 ± 0.009	1458.249	<1.00E-300

Table S1. Nutrient profiles per country, standardized by total calorie intake

Parameter	Japan	Mexico	Spain	UK	US	Statistic	Q-value
Ketchup	0.000 ± 0.001	0.001 ± 0.003	0.000 ± 0.001	0.001 ± 0.003	0.001 ± 0.004	20.244	0.000447
100% Fruit Juice such as Apple, Grape and Cran...	0.003 ± 0.016	0.001 ± 0.007	0.002 ± 0.008	0.004 ± 0.019	0.005 ± 0.021	25.04	5.02E-05
Corn, Soybean, Peanut, Sesame and Sunflower Oil	0.001 ± 0.002	0.001 ± 0.002	0.000 ± 0.001	0.000 ± 0.000	0.000 ± 0.000	26.626	2.45E-05
Margarine	0.001 ± 0.003	0.000 ± 0.001	0.000 ± 0.001	0.001 ± 0.002	0.000 ± 0.001	36.031	3.00E-07
Eggs	0.021 ± 0.039	0.021 ± 0.022	0.015 ± 0.012	0.015 ± 0.019	0.016 ± 0.023	37.249	1.72E-07
Jam, Jelly, Honey, Syrup or Sugar	0.002 ± 0.004	0.003 ± 0.006	0.002 ± 0.004	0.002 ± 0.004	0.002 ± 0.004	41.37	2.46E-08
Oranges, Grapefruit and Tangerines (Not Juice)	0.015 ± 0.026	0.015 ± 0.030	0.020 ± 0.030	0.020 ± 0.035	0.015 ± 0.026	46.12	2.58E-09
Potato, Tortilla Chips, Corn Chips and Puffs	0.004 ± 0.006	0.002 ± 0.005	0.002 ± 0.003	0.003 ± 0.005	0.004 ± 0.006	46.65	2.04E-09
Beer	0.043 ± 0.100	0.034 ± 0.083	0.026 ± 0.040	0.025 ± 0.071	0.037 ± 0.080	48.015	1.08E-09
Bacon and Breakfast Sausage	0.001 ± 0.002	0.001 ± 0.001	0.001 ± 0.002	0.001 ± 0.003	0.001 ± 0.002	63.62	5.91E-13
Grapes	0.003 ± 0.004	0.005 ± 0.008	0.010 ± 0.015	0.008 ± 0.015	0.005 ± 0.009	76.163	1.36E-15
Liquor and Mixed Drinks	0.006 ± 0.019	0.002 ± 0.007	0.000 ± 0.001	0.003 ± 0.009	0.004 ± 0.010	97.155	4.86E-20
Coffee	0.096 ± 0.117	0.091 ± 0.160	0.029 ± 0.031	0.119 ± 0.196	0.128 ± 0.184	97.42	4.35E-20
Cookies and Cakes	0.003 ± 0.004	0.004 ± 0.005	0.002 ± 0.005	0.005 ± 0.010	0.004 ± 0.007	99.657	1.49E-20
Low and Nonfat Milk	0.017 ± 0.082	0.026 ± 0.068	0.049 ± 0.078	0.031 ± 0.055	0.021 ± 0.061	100.939	8.10E-21
100% Orange Juice and Grapefruit Juice	0.004 ± 0.011	0.009 ± 0.026	0.006 ± 0.017	0.008 ± 0.023	0.004 ± 0.017	106.754	4.78E-22
Apples, Applesauce and Pears	0.011 ± 0.016	0.023 ± 0.028	0.027 ± 0.034	0.034 ± 0.039	0.023 ± 0.029	119.045	1.17E-24
Organ Meats	0.001 ± 0.001	0.000 ± 0.001	0.001 ± 0.005	0.000 ± 0.002	0.000 ± 0.001	125.331	5.41E-26
Onions and Leeks	0.019 ± 0.020	0.012 ± 0.015	0.022 ± 0.021	0.022 ± 0.022	0.016 ± 0.019	127.109	2.31E-26
Bananas	0.009 ± 0.015	0.018 ± 0.019	0.019 ± 0.019	0.021 ± 0.023	0.019 ± 0.025	127.654	1.81E-26
Ice Cream and Milkshakes	0.009 ± 0.012	0.004 ± 0.009	0.005 ± 0.016	0.005 ± 0.010	0.009 ± 0.016	129.092	9.14E-27
Canned Fish and Shellfish	0.003 ± 0.004	0.006 ± 0.010	0.006 ± 0.007	0.004 ± 0.006	0.005 ± 0.008	134.626	6.14E-28
Sweets and Confectionery	0.005 ± 0.007	0.002 ± 0.004	0.004 ± 0.006	0.005 ± 0.006	0.003 ± 0.005	142.877	1.08E-29
Carrots and Winter Squash	0.011 ± 0.013	0.012 ± 0.015	0.015 ± 0.015	0.026 ± 0.027	0.019 ± 0.025	172.248	5.58E-36
Mayonnaise	0.001 ± 0.003	0.001 ± 0.002	0.000 ± 0.001	0.001 ± 0.002	0.001 ± 0.002	179.048	1.99E-37
Red Wine	0.006 ± 0.025	0.011 ± 0.026	0.004 ± 0.008	0.025 ± 0.045	0.014 ± 0.037	186.072	6.34E-39
Lunch Meats	0.001 ± 0.002	0.005 ± 0.007	0.003 ± 0.004	0.002 ± 0.004	0.003 ± 0.007	189.404	1.26E-39
White, Rosé, Muscatel and Rice Wine	0.002 ± 0.009	0.002 ± 0.006	0.003 ± 0.007	0.014 ± 0.030	0.009 ± 0.026	197.939	1.89E-41
Leafy Greens	0.006 ± 0.006	0.015 ± 0.026	0.019 ± 0.019	0.018 ± 0.021	0.021 ± 0.024	217.557	1.18E-45
Regular Soft Drinks (Not Diet)	0.042 ± 0.114	0.021 ± 0.055	0.005 ± 0.027	0.002 ± 0.010	0.007 ± 0.049	222.501	1.05E-46
Commercial Fruit Juices	0.020 ± 0.038	0.000 ± 0.003	0.003 ± 0.021	0.001 ± 0.007	0.004 ± 0.022	239.099	2.90E-50
Berries such as Strawberries and Blueberries	0.004 ± 0.006	0.011 ± 0.018	0.004 ± 0.004	0.028 ± 0.038	0.026 ± 0.039	244.292	2.28E-51
Cheese	0.005 ± 0.011	0.008 ± 0.009	0.016 ± 0.013	0.011 ± 0.012	0.012 ± 0.014	248.566	2.84E-52
Shellfish	0.002 ± 0.002	0.002 ± 0.003	0.004 ± 0.003	0.001 ± 0.003	0.001 ± 0.003	265.597	6.30E-56
Mushrooms	0.006 ± 0.006	0.004 ± 0.006	0.014 ± 0.017	0.008 ± 0.010	0.005 ± 0.009	277.964	1.41E-58
Butter or Ghee	0.001 ± 0.004	0.001 ± 0.003	0.001 ± 0.001	0.003 ± 0.004	0.003 ± 0.003	290.597	2.77E-61
Muffins, Scones, Croissants and Biscuits	0.004 ± 0.006	0.007 ± 0.011	0.007 ± 0.010	0.004 ± 0.008	0.002 ± 0.004	290.95	2.43E-61
Yogurt	0.099 ± 0.156	0.018 ± 0.026	0.027 ± 0.032	0.030 ± 0.034	0.019 ± 0.029	294.359	4.66E-62
Cruciferous Vegetables	0.025 ± 0.025	0.010 ± 0.017	0.017 ± 0.022	0.028 ± 0.028	0.022 ± 0.029	295.242	3.14E-62
Watermelon and Red Melon	0.002 ± 0.005	0.005 ± 0.013	0.009 ± 0.014	0.001 ± 0.004	0.004 ± 0.014	321.672	6.53E-68
Cherries, Picotas, Plums, Peaches, Nectarines,...	0.001 ± 0.002	0.005 ± 0.016	0.015 ± 0.018	0.004 ± 0.011	0.005 ± 0.013	328.598	2.19E-69
White Fish	0.007 ± 0.012	0.005 ± 0.010	0.012 ± 0.019	0.005 ± 0.008	0.003 ± 0.008	335.745	6.62E-71
Whole Milk	0.074 ± 0.166	0.027 ± 0.063	0.017 ± 0.048	0.012 ± 0.034	0.008 ± 0.031	346.447	3.42E-73
Summer Squash, Zucchini, Eggplants, Cucumbers	0.006 ± 0.009	0.007 ± 0.014	0.017 ± 0.017	0.007 ± 0.012	0.007 ± 0.012	349.74	7.04E-74
Potatoes	0.005 ± 0.005	0.008 ± 0.010	0.019 ± 0.016	0.024 ± 0.026	0.012 ± 0.023	364.756	4.28E-77
Dark Fish	0.008 ± 0.008	0.003 ± 0.007	0.011 ± 0.010	0.008 ± 0.010	0.005 ± 0.008	374.808	3.08E-79
Peppers Cooked and Raw	0.002 ± 0.002	0.011 ± 0.019	0.015 ± 0.015	0.019 ± 0.022	0.013 ± 0.021	384.82	2.27E-81
Tea	0.085 ± 0.116	0.075 ± 0.166	0.015 ± 0.026	0.309 ± 0.287	0.113 ± 0.183	391.844	7.42E-83
Processed Meats	0.003 ± 0.009	0.001 ± 0.002	0.002 ± 0.003	0.000 ± 0.002	0.001 ± 0.004	422.577	1.84E-89
Chicken and Turkey	0.011 ± 0.011	0.011 ± 0.012	0.033 ± 0.027	0.008 ± 0.010	0.011 ± 0.014	423.204	1.47E-89
Garlic	0.000 ± 0.000	0.001 ± 0.001	0.000 ± 0.000	0.001 ± 0.001	0.001 ± 0.001	444.895	3.31E-94
Fresh Tomatoes	0.007 ± 0.011	0.029 ± 0.035	0.038 ± 0.032	0.036 ± 0.040	0.018 ± 0.022	467.956	3.80E-99
Beef, Pork, Ham and Lamb	0.025 ± 0.045	0.015 ± 0.015	0.022 ± 0.017	0.007 ± 0.011	0.007 ± 0.012	519.566	2.94E-110
Green or String Beans	0.000 ± 0.001	0.003 ± 0.006	0.015 ± 0.015	0.007 ± 0.012	0.004 ± 0.008	560.016	5.97E-119
White Breads	0.030 ± 0.049	0.004 ± 0.007	0.009 ± 0.014	0.005 ± 0.010	0.004 ± 0.010	565.587	4.34E-120
Peanut Butter, Peanuts and Other Nuts and Seeds	0.001 ± 0.005	0.001 ± 0.003	0.013 ± 0.012	0.007 ± 0.011	0.009 ± 0.010	738.181	2.26E-157
Olive Oil	0.001 ± 0.002	0.002 ± 0.004	0.012 ± 0.008	0.004 ± 0.006	0.004 ± 0.007	824.332	6.18E-176
Soups and Creams	0.067 ± 0.060	0.045 ± 0.040	0.002 ± 0.004	0.029 ± 0.039	0.034 ± 0.045	861.841	6.17E-184
Salt	0.002 ± 0.001	0.000 ± 0.001	0.001 ± 0.001	0.000 ± 0.001	0.000 ± 0.001	885.491	6.95E-189
Rice, Pasta and Noodles	0.053 ± 0.044	0.000 ± 0.001	0.015 ± 0.013	0.015 ± 0.014	0.019 ± 0.021	1006.472	8.47E-215

Table S2. Food profiles per country, standardized by total calorie intake

Enriched Taxa	Coverage	Japan CLR	Japan RA	Japan Prev	Mexico CLR	Mexico RA	Mexico Prev	Spain CLR	Spain RA	Spain Prev	UK CLR	UK RA	UK Prev	US CLR	US RA	US Prev
s__Bacillus subtilis;G000747645	99.7316	1.4576	0.0001	0.6585	-2.3700	0.0000	0.0907	-2.2810	0.0000	0.0644	-2.0066	0.0000	0.0682	-1.9552	0.0000	0.0827
s__Bacillus subtilis;G000696615	85.1857	1.1292	0.0001	0.6317	-2.3690	0.0000	0.0882	-2.2881	0.0000	0.0578	-2.0019	0.0000	0.0747	-1.9599	0.0000	0.0803
s__Bacillus subtilis;G900110305	83.0138	1.1149	0.0001	0.6317	-2.3683	0.0000	0.0907	-2.2877	0.0000	0.0600	-2.0046	0.0000	0.0682	-1.9560	0.0000	0.0754
s__Bacillus spizizenii;G000740655	42.9593	0.1100	0.0000	0.5195	-2.4241	0.0000	0.0050	-2.3301	0.0000	0.0200	-2.0513	0.0000	0.0292	-1.9459	0.0000	0.0268
s__Bacillus cabrialesii;G001663575	38.6412	0.0492	0.0000	0.4902	-2.4182	0.0000	0.0101	-2.3257	0.0000	0.0133	-2.0616	0.0000	0.0162	-1.9458	0.0000	0.0268
s__Bacillus velezensis;G001687195	4.5847	-1.0408	0.0000	0.3585	-2.4214	0.0000	0.0101	-2.3287	0.0000	0.0222	-2.0852	0.0000	0.0227	-1.9794	0.0000	0.0292
s__Bacillus amyloliquefaciens;G000196735	3.9993	-1.3319	0.0000	0.3268	-2.4205	0.0000	0.0252	-2.3177	0.0000	0.0511	-2.0954	0.0000	0.0195	-2.0015	0.0000	0.0341
s__Bacillus velezensis;G001461825	4.4335	-1.3784	0.0000	0.3000	-2.4238	0.0000	0.0126	-2.3380	0.0000	0.0200	-2.0880	0.0000	0.0162	-1.9751	0.0000	0.0268
s__Bacillus velezensis;G001517185	4.3095	-1.4982	0.0000	0.2732	-2.4244	0.0000	0.0050	-2.3298	0.0000	0.0178	-2.0848	0.0000	0.0130	-1.9781	0.0000	0.0219
s__Bacillus velezensis;G001617995	4.4580	-1.4749	0.0000	0.2732	-2.4250	0.0000	0.0025	-2.3358	0.0000	0.0156	-2.0870	0.0000	0.0065	-1.9790	0.0000	0.0243
s__Bacillus velezensis;G001587325	4.1379	-1.5126	0.0000	0.2683	-2.4239	0.0000	0.0050	-2.3371	0.0000	0.0200	-2.0870	0.0000	0.0065	-1.9823	0.0000	0.0268
s__Bacillus velezensis;G900156245	4.3072	-1.5450	0.0000	0.2585	-2.4252	0.0000	0.0050	-2.3352	0.0000	0.0133	-2.0839	0.0000	0.0065	-1.9784	0.0000	0.0219
s__Bacillus velezensis;G000740715	4.3269	-1.5182	0.0000	0.2537	-2.4269	0.0000	0.0050	-2.3340	0.0000	0.0133	-2.0865	0.0000	0.0032	-1.9757	0.0000	0.0243
s__Bacillus velezensis;G001440465	4.4382	-1.6177	0.0000	0.2463	-2.4252	0.0000	0.0076	-2.3351	0.0000	0.0111	-2.0855	0.0000	0.0032	-1.9674	0.0000	0.0243
s__Bacillus velezensis;G001975995	4.0183	-1.6084	0.0000	0.2463	-2.4241	0.0000	0.0076	-2.3352	0.0000	0.0133	-2.0865	0.0000	0.0032	-1.9752	0.0000	0.0243
s__Bacillus velezensis;G000746035	4.3111	-1.5791	0.0000	0.2439	-2.4250	0.0000	0.0025	-2.3346	0.0000	0.0178	-2.0870	0.0000	0.0065	-1.9802	0.0000	0.0268
s__Bacillus glycinifermentans;G900093775	1.2710	-1.6290	0.0000	0.2341	-2.3993	0.0000	0.0327	-2.3383	0.0000	0.0244	-2.0838	0.0000	0.0227	-1.9977	0.0000	0.0122
s__Bacillus pumilus;G001578205	1.1230	-1.7973	0.0000	0.1829	-2.4236	0.0000	0.0076	-2.3414	0.0000	0.0178	-2.0879	0.0000	0.0130	-1.9978	0.0000	0.0073
s__Novosphingobium nitrogenifigens;G000192575	1.8637	-1.7017	0.0000	0.2049	-2.3780	0.0000	0.0529	-2.3042	0.0000	0.0556	-2.0643	0.0000	0.0227	-1.9551	0.0000	0.0487
s__Agrobacterium arsenijevicii;G000949895	12.9251	-1.9587	0.0000	0.0561	-1.4681	0.0000	0.5189	-2.2592	0.0000	0.1044	-2.0816	0.0000	0.0325	-1.9649	0.0000	0.0681
s__Limnospira fusiformis;G000307915	76.3473	-1.9267	0.0000	0.0512	-1.2238	0.0000	0.5567	-2.1141	0.0000	0.1733	-1.8774	0.0000	0.0974	-1.8552	0.0000	0.0876
s__Enterococcus_B faecalis_A;G000415185	1.5150	-1.9185	0.0000	0.0659	-1.9288	0.0000	0.2997	-2.2835	0.0000	0.0578	-2.0481	0.0000	0.0325	-1.9694	0.0000	0.0341
s__Enterococcus_J nangangensis;G005405245	1.0542	-1.9681	0.0000	0.0585	-2.0971	0.0000	0.3048	-2.3082	0.0000	0.0800	-2.0847	0.0000	0.0552	-1.9807	0.0000	0.0511
s__Enterococcus_A hermanniensis;G001885945	2.9648	-1.8502	0.0000	0.1024	-1.9333	0.0000	0.3123	-2.2937	0.0000	0.0667	-2.0472	0.0000	0.0519	-1.9638	0.0000	0.0389
s__Atopococcus tabaci;G000429585	1.2611	-1.9910	0.0000	0.0317	-2.1564	0.0000	0.2267	-2.3139	0.0000	0.0422	-2.0496	0.0000	0.0390	-1.9608	0.0000	0.0511
s__Lacticaseibacillus pantheris;G001311175	69.9422	-1.9964	0.0000	0.0439	-2.1429	0.0000	0.2292	-2.3052	0.0000	0.0689	-2.0882	0.0000	0.0227	-1.9642	0.0000	0.0511
s__Lacticaseibacillus pantheris;G001435155	69.8604	-2.0031	0.0000	0.0366	-2.1393	0.0000	0.2317	-2.3003	0.0000	0.0756	-2.0872	0.0000	0.0260	-1.9651	0.0000	0.0487
s__Weissella minor;G001437425	1.2448	-2.0314	0.0000	0.0073	-2.2759	0.0000	0.1209	-2.3109	0.0000	0.0311	-2.0796	0.0000	0.0065	-1.9992	0.0000	0.0049
s__Companilactobacillus farciminis;G000184535	2.7907	-2.0221	0.0000	0.0122	-2.3015	0.0000	0.0882	-2.3385	0.0000	0.0067	-2.0879	0.0000	0.0130	-1.9927	0.0000	0.0243
s__Ochrobactrum anthropi;G001586735	6.1953	-2.0167	0.0000	0.0195	-2.3736	0.0000	0.0756	-2.3299	0.0000	0.0222	-2.0911	0.0000	0.0032	-2.0008	0.0000	0.0195
s__UBA1436 sp902794895;G902794895	1.0240	-2.0203	0.0000	0.0024	-2.1657	0.0000	0.0605	-2.2712	0.0000	0.0133	-2.0695	0.0000	0.0097	-1.9906	0.0000	0.0024
s__Helicobacter_D apodemus;G000765745	1.0037	-2.0335	0.0000	0.0000	-2.3680	0.0000	0.0403	-2.3359	0.0000	0.0000	-2.0909	0.0000	0.0000	-2.0036	0.0000	0.0024
s__GCF-000787395 sp000787395;G002021855	1.0401	-2.0130	0.0000	0.0073	-2.3414	0.0000	0.0428	-2.3367	0.0000	0.0022	-2.0956	0.0000	0.0032	-2.0036	0.0000	0.0049
s__Acinetobacter marinus;G900096915	2.2366	-2.0236	0.0000	0.0073	-2.3672	0.0000	0.0479	-2.3331	0.0000	0.0156	-2.0806	0.0000	0.0130	-2.0007	0.0000	0.0146
s__Salinivibrio socompensis;G000513715	1.4755	-2.0118	0.0000	0.0098	-2.3696	0.0000	0.0428	-2.3331	0.0000	0.0089	-2.0975	0.0000	0.0130	-1.9954	0.0000	0.0049
s__Aliiglaciecola lipolytica;G000314975	1.1958	-2.0096	0.0000	0.0049	-2.4099	0.0000	0.0252	-2.3251	0.0000	0.0044	-2.0933	0.0000	0.0032	-1.9984	0.0000	0.0073
s__GCF-000787395 sp000787395;G002021105	1.2291	-2.0186	0.0000	0.0024	-2.4232	0.0000	0.0101	-2.3352	0.0000	0.0022	-2.0963	0.0000	0.0032	-2.0019	0.0000	0.0000
s__Bdellovibrio bacteriovorus_F;G000317895	3.0394	-2.0335	0.0000	0.0000	-2.3841	0.0000	0.0428	-2.0693	0.0000	0.1578	-2.0171	0.0000	0.0422	-1.9843	0.0000	0.0243
s__Bdellovibrio bacteriovorus;G000196175	4.2009	-2.0335	0.0000	0.0000	-2.3783	0.0000	0.0453	-2.0355	0.0000	0.1622	-2.0330	0.0000	0.0487	-1.9890	0.0000	0.0243
s__Bdellovibrio bacteriovorus;G000691605	4.0053	-2.0335	0.0000	0.0000	-2.3882	0.0000	0.0353	-2.0410	0.0000	0.1622	-2.0304	0.0000	0.0519	-1.9913	0.0000	0.0243
s__Sediminibacterium sp000515315;G000515315	1.2030	-2.0364	0.0000	0.0024	-2.4253	0.0000	0.0000	-2.2532	0.0000	0.1089	-2.0912	0.0000	0.0065	-1.9998	0.0000	0.0073
s__GCA-2401445 sp002401445;G002401445	1.1152	-2.0316	0.0000	0.0024	-2.4257	0.0000	0.0025	-2.3034	0.0000	0.0533	-2.0891	0.0000	0.0032	-2.0019	0.0000	0.0000
s__Methanoculleus sp002508705;G002508805	76.8821	-2.0335	0.0000	0.0000	-2.4253	0.0000	0.0000	-2.3359	0.0000	0.0000	-2.0318	0.0000	0.0162	-1.9997	0.0000	0.0024
s__;G900660535	1.2755	-2.0335	0.0000	0.0000	-2.4237	0.0000	0.0025	-2.3355	0.0000	0.0044	-2.0534	0.0000	0.0162	-2.0023	0.0000	0.0024
s__Buchnera aphidicola_A;G000225445	2.8668	-2.0311	0.0000	0.0024	-2.4126	0.0000	0.0025	-2.3359	0.0000	0.0000	-2.0975	0.0000	0.0162	-2.0071	0.0000	0.0049
s__Moheibacter sediminis;G900176425	1.4798	-2.0258	0.0000	0.0171	-2.4027	0.0000	0.0479	-2.3347	0.0000	0.0289	-2.0744	0.0000	0.0455	-1.9516	0.0000	0.1582
s__Soonwooa buanensis;G900167905	1.5106	-1.9292	0.0000	0.0366	-2.4064	0.0000	0.0277	-2.3419	0.0000	0.0200	-2.0928	0.0000	0.0325	-2.0038	0.0000	0.1144
s__Aliarcobacter faecis;G000585155	1.0582	-2.0335	0.0000	0.0000	-2.4174	0.0000	0.0076	-2.3381	0.0000	0.0044	-2.0884	0.0000	0.0065	-1.9734	0.0000	0.0243

Table S3. Genome Coverage, Mean CLR, Mean Relative Abundance, and Prevalence of Enriched Taxa Per Country
