# Supplementary material for: Five-country analysis of geographic and dietary drivers of gut microbiome composition and genomic variation
Source: Nat Commun. 2026 Sep 21;17:11004. doi: 10.1038/s41467-026-77928-9 (PMC13687547; doi:10.1038/s41467-026-77928-9)
Supplement: Supplementary file 3 [file 41467_2026_77928_MOESM3_ESM.pdf]

nature portfolio

Peer Review File

Five-country analysis of geographic and dietary drivers of gut microbiome composition and genomic variation

Corresponding Author: Dr Andrew Bartko

This file contains all reviewer reports in order by version, followed by all author rebuttals in order by version.

Version 0:

Reviewer comments:

Reviewer #1

(Remarks to the Author)
This manuscript presents a large and valuable multi-country dataset integrating dietary metadata, lifestyle variables, shotgun metagenomics, and genome-resolved analyses across nearly 2,000 individuals. The study addresses an important question regarding the universality against context-dependence of microbiome–diet relationships and has clear potential to become a reference for the field. The dataset itself is impressive and the analytical goal ambitious.
However, in its current form, the manuscript does not yet fully capitalise on the potential of the dataset. Some aspects of the microbiome data processing and interpretation are insufficiently explained or justified, and some choices appear underdeveloped. Clarifying these points and strengthening methodological transparency would increase the impact and robustness of the study.

Specific comments:

- Unclear definition and use of microbiome data types. The manuscript refers interchangeably to “features,” “taxa,” “OGUs,” genomes, and MAG-derived species bins without clearly explaining how each data layer was generated or which analyses use which representation. A schematic overview (raw reads, OGUs, taxa, MAGs, gene-level analyses) would greatly improve clarity and reproducibility. For instance, the authors refer to “multiple Escherichia coli OGUs,” but no formal definition or methodological description of OGUs is provided in either Results or Methods. Since OGUs are not a universally standardised unit across microbiome studies, the manuscript must explicitly state what they represent, how they were generated, and how they relate to reference genomes and taxonomic units.
- Sequencing depth reporting is insufficient. The manuscript reports only a minimum read-depth filter (>1,000,000 reads per sample) but does not provide summary statistics (mean, median, distribution) of sequencing depth across samples or countries. These values are important to evaluate comparability across cohorts and to assess whether geographic clustering could reflect depth variation rather than biological signal.
- Threshold of ≥30 MAGs per species may bias results. Restricting analyses to species represented by ≥30 MAGs may preferentially retain abundant or easily assembled taxa while excluding biologically relevant but lower-coverage species. This threshold appears arbitrary and may reflect sequencing depth or assembly efficiency rather than biological prevalence. The authors should justify this choice or perform sensitivity analyses with alternative thresholds. A suggestion in this direction could be to construct a species-level genome bin (SGB) catalog from dereplicated MAGs and map reads from each metagenome back to this catalog to quantify abundance and coverage across samples. This would allow filtering species based on true prevalence rather than assembly success and would strengthen conclusions regarding geographic structure.
- Limited justification for species selected for gene-level analysis. The manuscript presents gene presence/absence analyses for Bifidobacterium longum and Faecalibacterium longum, but it is unclear why these species were selected. The authors should specify whether selection was hypothesis-driven (e.g., prevalence, biological relevance, genome quality) or post hoc based on observed signals, and whether similar patterns are observed across other species.
- Genome-resolved analysis section underdeveloped. The strain-level and gene-level analyses and their connection with diet are potentially among the most novel components of the manuscript, yet they are comparatively brief and lack depth relative to their importance. Additional methodological detail, broader validation across taxa, and clearer integration with the study’s central claims would strengthen this section considerably.
- Interpretation occasionally exceeds evidence. Some interpretations occasionally exceed the strength of the presented evidence. For example, genomic differentiation is described as reflecting localised selective pressures, yet the analyses do not directly test evolutionary selection. Similarly, conclusions regarding diet shaping microbiome structure appear stronger

than warranted by cross-sectional correlation analyses. These statements should be rephrased to emphasise association rather than causation unless additional analyses are provided.

(Remarks on code availability)

Reviewer #2

(Remarks to the Author)
The manuscript “The Human Diets and Microbiome Initiative: A five-country analysis of geographic and dietary drivers of gut microbiome composition and genomic variation” is a novel and well written study, representing a significant collaborative effort. The authors have done a wonderful job of handling what must have been a truly massive dataset.

Major/General Comments:

1. Since the study design is not balanced for age, sex or bmi across countries, how can the authors be sure that findings regarding geography and host diet – microbiome relationships are not confounded by these variables? It might be interesting to draw more limited conclusions from this dataset with higher confidence. For example, are the observations robust if you look at only females across the five countries, or does the cosmetics finding disappear? Do the same country specific microbiome-HEI score relationships remain if the dataset is stratified by BMI?

2. The claims regarding standardization of methods to reduce technical variation are very strong. Is there a demonstration that this standardization of methods was effective. For example was an analysis of mock communities received from each country performed?
Minor/Specific Comments:

3. Line 111: Japan vs..?

4. Lines 107 – 130 and Figure 1:
a. One of the top 10 metadata features contributing to both PC1 and PC2 is Sex. And Table 1 shows that there were significant differences in study subject sex by country. Could the differences observed in cosmetics/supplement use by country be driven by sex differences in participants within countries?
b. The remaining top 10 metadata variables driving variation across PC1 are all BMI related. The methods describe great care that was taken to manage the high number of variables in this dataset. However, is it possible that some additional variable reduction regarding body weight/size could (should?) have taken place prior to performing this analysis.

5. Lines 231 – 233: Fermented foods can be difficult to classify in food records. Can information on how and why the particular foods were included/excluded for the analysis shown in Figure S6 be added to the methods?

6. Line 245: Mash?

7. Line 418: What does the BCL acronym stand for?

8. Line 491: Reference for gemelli?

9. What was the rationale for artificially reducing variation within a given variable? For example, the home cooked meals frequency? It seems like retaining the variation in the number of home cooked meals reported by individuals would benefit the multivariate nature of the analysis performed.

(Remarks on code availability)

Version 1:

Reviewer comments:

Reviewer #1

(Remarks to the Author)
The revised manuscript is substantially improved and addresses the major concerns raised in the original review. The clarification of microbiome data representations, additional sequencing-depth analyses, expanded genome-resolved analyses, and more cautious interpretation significantly strengthen the work. I have only one remaining suggestion. While I appreciate the authors' justification for the ≥30 MAG threshold in terms of statistical power, I still believe that read mapping against representative MAGs or species-level genome bins would provide valuable complementary information regarding biological prevalence and would help contextualize the observed genomic differentiation. Aside from this point, I am satisfied with the revision.

Specific comment

- Biological prevalence versus MAG recovery.
I appreciate the authors' justification for the ≥30 MAG threshold in terms of statistical power for genome-resolved analyses. The explanation clarifies why this threshold is necessary for the downstream PERMANOVA and GWAS approaches. However, this does not fully address the original concern that MAG recovery is influenced by assembly success, sequencing depth, and genome characteristics, and therefore does not necessarily reflect the true biological prevalence of a species across populations. For this reason, I still believe that mapping metagenomic reads back to representative MAGs or species-level genome bins would provide valuable complementary information on species prevalence and abundance across cohorts. Such an analysis would not replace the genome-resolved approach, but rather complement it by providing an additional biological context for interpreting the observed genomic differentiation. This would help readers assess whether the strongest genomic signals are associated with widespread and abundant taxa or are restricted to a smaller subset of the microbiome.

(Remarks on code availability)

Reviewer #2

(Remarks to the Author)
The authors have sufficiently addressed all of my concerns.

(Remarks on code availability)

Open Access This Peer Review File is licensed under a Creative Commons Attribution 4.0 International License, which permits use, sharing, adaptation, distribution and reproduction in any medium or format, as long as you give appropriate credit to the original author(s) and the source, provide a link to the Creative Commons license, and indicate if changes were made.
In cases where reviewers are anonymous, credit should be given to 'Anonymous Referee' and the source.
The images or other third party material in this Peer Review File are included in the article’s Creative Commons license, unless indicated otherwise in a credit line to the material. If material is not included in the article’s Creative Commons license and your intended use is not permitted by statutory regulation or exceeds the permitted use, you will need to obtain permission directly from the copyright holder.
To view a copy of this license, visit https://creativecommons.org/licenses/by/4.0/

Response to Reviewers

Title: The Human Diets & Microbiome Initiative: A five-country analysis of geographic and dietary drivers of gut microbiome composition and genomic variation

General Response

We thank the editor and reviewers for their thoughtful and constructive feedback. We are pleased that the reviewers recognized the value of the dataset and the potential of this work as a reference for the field. We have carefully revised the manuscript to address all comments raised. All changes are highlighted in the revised manuscript. Below, we provide a point-by-point response to each reviewer comment.

Reviewer #1

We thank Reviewer #1 for their insightful comments and for recognizing the value and ambition of this dataset. We have addressed all concerns as detailed below.

Comment 1.1

"Unclear definition and use of microbiome data types. The manuscript refers interchangeably to "features," "taxa," "OGUs," genomes, and MAG-derived species bins without clearly explaining how each data layer was generated or which analyses use which representation. A schematic overview (raw reads, OGUs, taxa, MAGs, gene-level analyses) would greatly improve clarity and reproducibility. For instance, the authors refer to "multiple Escherichia coli OGUs," but no formal definition or methodological description of OGUs is provided in either Results or Methods. Since OGUs are not a universally standardised unit across microbiome studies, the manuscript must explicitly state what they represent, how they were generated, and how they relate to reference genomes and taxonomic units."

Response:
We thank the reviewer for this important comment and agree that clearer and more consistent definitions of microbiome data representations are essential. To address this, we have made several revisions to improve clarity, consistency, and reproducibility throughout the manuscript.

1. We standardized terminology by replacing ambiguous terms such as "features" and "taxa" with precise definitions of the specific data representations used. In particular, we now consistently refer to Operational Genomic Units (OGUs) when describing reference genome–aligned features, and explicitly distinguish these from genome-resolved units.
2. We have added a new schematic overview (Supplementary Fig. 1) illustrating the full analytical metagenomic workflow, from raw sequencing reads through quality control, OGU generation, MAG-derived species bins construction, as well as their respective downstream analyses. This figure clarifies how each data layer is generated and which analyses use each representation.
3. We have expanded the Methods (pages 14-15) to provide explicit definitions and descriptions of all microbiome data types used in this study, including: Operational Genomic Units (OGUs), Metagenome-assembled genomes (MAGs), and species-level bins.

Comment 1.2

"Sequencing depth reporting is insufficient. The manuscript reports only a minimum read-depth filter (>1,000,000 reads per sample) but does not provide summary statistics (mean, median, distribution) of sequencing depth across samples or countries. These values are important to evaluate comparability across cohorts and to assess whether geographic clustering could reflect depth variation rather than biological signal."

Response:

We thank the reviewer for this suggestion. We have now added summary statistics of sequencing depth across samples, including mean and standard deviation stratified by country in Table 1. As noted by the reviewer, sequencing depth varies across countries. We have added this discrepancy in the first paragraph of the results section on page 4.

To assess whether sequencing depth could confound observed geographic clustering, we evaluated the contribution of sequencing depth to microbiome variation by including it as a covariate in the PERMANOVA model. We found that geographic differences remained significant after accounting for sequencing depth, and that sequencing depth explained substantially less variation than country (Results: page 5, Methods: page 15). These additions allow assessment of comparability across cohorts and evaluation of potential confounding by sequencing depth.

Comment 1.3

"Threshold of ≥30 MAGs per species may bias results. Restricting analyses to species represented by ≥30 MAGs may preferentially retain abundant or easily assembled taxa while excluding biologically relevant but lower-coverage species. This threshold appears arbitrary and may reflect sequencing depth or assembly efficiency rather than biological prevalence. The authors should justify this choice or perform sensitivity analyses with alternative thresholds. A suggestion in this direction could be to construct a species-level genome bin (SGB) catalog from dereplicated MAGs and map reads from each metagenome back to this catalog to quantify abundance and coverage across samples. This would allow filtering species based on true prevalence rather than assembly success and would strengthen conclusions regarding geographic structure."

Response:

We thank the reviewer for this insightful comment. We agree that restricting our analysis to species with ≥30 high-quality MAGs inherently biases our dataset toward more abundant and easily assembled taxa, and we acknowledge that read-mapping approaches provide a superior metric for true biological prevalence. However, we have retained our assembly-based threshold for this specific analysis for two primary reasons:

1. *The research question requires assembly-based resolution.* While read-mapping to a dereplicated SGB catalog is the gold standard for quantifying species abundance and prevalence, our study aims to evaluate the geographic stratification of strain-level genetic architecture. Mapping reads to a representative consensus SGB inherently masks individual-specific accessory gene content, structural variations, and gene gain/loss events. Because the geographic structure we observe is driven substantially by this accessory content, relying on read-mapping would obscure the biological signals we are measuring.
2. *Statistical power constraints dictate the threshold.* The threshold of 30 MAGs was not chosen to represent biological prevalence, but rather to meet the mathematical prerequisites for our

statistical models. To perform robust population genetics testing (e.g., PERMANOVA) and pangenome-wide association studies across five distinct countries, we required a sample size that provides adequate degrees of freedom (averaging ≥6 independent genomes per geographic cohort). Dropping below this threshold compromises the statistical power required to detect significant geographic stratification. We have added this justification to the Results and Methods section (pages 7 & 17, respectively).

Comment 1.4

"Limited justification for species selected for gene-level analysis. The manuscript presents gene presence/absence analyses for Bifidobacterium longum and Faecalibacterium longum, but it is unclear why these species were selected. The authors should specify whether selection was hypothesis-driven (e.g., prevalence, biological relevance, genome quality) or post hoc based on observed signals, and whether similar patterns are observed across other species."

Response:

To investigate finer-resolution patterns, we selected two species with high MAG recovery across all five countries and significant PERMANOVA results for gene presence/absence analysis. These species serve as examples of a broader pattern. We have added this justification to the Results and Methods section (page 8 & 18, respectively). To demonstrate generalizability, we added a cross-species KEGG pathway enrichment analysis spanning all significantly differentiated species (revised Figure 4C), showing that carbohydrate metabolism, amino acid biosynthesis, and cofactor production pathways are recurrently differentiated across independent lineages. We have also refactored Supplemental Figure 8a to capture more interpretable KEGG pathway differences across species and countries, and created Supplemental Table 5 summarizing KEGG categories enriched or depleted across all species from all countries with sufficient data for GWAS.

Comment 1.5

"Genome-resolved analysis section underdeveloped. The strain-level and gene-level analyses and their connection with diet are potentially among the most novel components of the manuscript, yet they are comparatively brief and lack depth relative to their importance. Additional methodological detail, broader validation across taxa, and clearer integration with the study's central claims would strengthen this section considerably."

Response:

We thank the reviewer for this suggestion. We expanded the genome-resolved section to strengthen breadth and rigor while highlighting potential links between diet and enrichment patterns. The key additions are:

1. A cross-species summary of gene-level differentiation (revised Figure 4b), which shows that the extent of geographically structured genes varies substantially across taxa;
2. A systematic cross-species pathway enrichment analysis (revised Figure 4c) revealing significantly over-represented KEGG pathways among geographically structured variants;
3. Comparisons between dietary intake and microbiome functional enrichment across countries (revised Figures 4d & S8b);

4. Identification of a dietary folate gradient that inversely tracks microbial folate biosynthesis gene enrichment across all five countries (revised Figure 4e);
5. Additional associations with dietary fat and vitamin B12, presented as hypothesis-generating observations (page 8); and
6. A more nuanced discussion acknowledging that while specific connections can be tied to measured dietary or demographic variables, the broader patterns of genomic differentiation likely reflect multiple factors including but not limited to diet (page 9).

This framing integrates the strain-level findings with the dietary data from Figures 1–3 and Tables S3–S4 while maintaining appropriate caution about causal inference from cross-sectional data.

Comment 1.6

"Interpretation occasionally exceeds evidence. Some interpretations occasionally exceed the strength of the presented evidence. For example, genomic differentiation is described as reflecting localised selective pressures, yet the analyses do not directly test evolutionary selection. Similarly, conclusions regarding diet shaping microbiome structure appear stronger than warranted by cross-sectional correlation analyses. These statements should be rephrased to emphasise association rather than causation unless additional analyses are provided."

Response:
We thank the reviewer for this important comment and agree that some interpretations in the original manuscript exceeded the strength of the evidence. To address this, we have revised the manuscript to ensure that all conclusions are appropriately framed as associations rather than causal relationships. Specifically, we have:

1. Replaced causal language (e.g., "shape," "reflect," "exert selective pressures") with more appropriate associative phrasing (e.g., "are associated with," "are consistent with," or "may contribute to") throughout the Abstract (page 3), Introduction (Page 4), Results (page 8-9), and Discussion (pages 8-10).
2. Revised descriptions of genomic differentiation to avoid implying evolutionary selection, instead framing these findings as patterns consistent with population-specific genomic variation (pages 10).
3. Softened statements regarding diet-microbiome relationships to emphasize that these findings arise from cross-sectional associations rather than direct causal effects (pages 9-10).
4. In addition, we strengthened the Discussion to explicitly acknowledge that causal mechanisms cannot be inferred from the current study design and that observed associations may reflect correlated environmental or lifestyle factors (page 11).

These revisions ensure that the interpretation of results is aligned with the analytical framework and evidence presented.

Reviewer #2

We thank Reviewer #2 for their positive assessment and constructive suggestions.

Comment 2.1
"Since the study design is not balanced for age, sex or BMI across countries, how can the authors be sure that findings regarding geography and host diet – microbiome relationships are not confounded by these variables? It might be interesting to draw more limited conclusions from this dataset with higher confidence. For example, are the observations robust if you look at only females across the five countries, or does the cosmetics finding disappear? Do the same country specific microbiome-HEI score relationships remain if the dataset is stratified by BMI?"

Response:
We thank the reviewer for this important point regarding potential confounding by demographic variables. We acknowledge that the study design is not fully balanced across countries with respect to age, sex, and BMI. To address this, we performed additional analyses to evaluate the robustness of our findings.

1. We incorporated age, sex, cosmetics frequency, and BMI as covariates in PERMANOVA models assessing associations between country and microbiome composition. Geographic differences remained highly significant after adjustment, with country explaining substantially more variation than these covariates (Results: page 5, Methods: page 15).
2. Additionally, we performed sensitivity analyses in subsets restricted to female participants and to individuals with BMI in the normal range. In both cases, geographic clustering remained significant, indicating that these patterns are not driven by sex or BMI differences.
3. With respect to microbiome–diet relationships, all HEI analyses were performed using models adjusted for age, sex, and BMI, and this has been clarified in the main text (page 6). We additionally explored stratified analyses (e.g., female-only and BMI-restricted subsets), which showed qualitatively similar patterns but reduced statistical significance, consistent with decreased sample size and statistical power. Given this loss of power and the consistency of covariate-adjusted models, we focused on adjusted analyses in the text.
4. Lastly, we have revised the Discussion to more clearly emphasize the limitations of causal interpretation, noting that observed associations may reflect unmeasured confounders or correlated environmental factors (page 11).

Comment 2.2
"The claims regarding standardization of methods to reduce technical variation are very strong. Is there a demonstration that this standardization of methods was effective. For example, was an analysis of mock communities received from each country performed?"

Response:
We thank the reviewer for this important point regarding the assessment of technical variation. We agree that demonstrating the effectiveness of methodological standardization is critical. While mock community controls were not included across sites, several aspects of the study design were implemented to minimize technical variability. Specifically, all samples were processed using a centralized and standardized workflow, including uniform DNA extraction protocols, sequencing on the same platform, and a consistent bioinformatics pipeline applied across all samples. To further evaluate the potential impact of technical variation, we assessed whether sequencing depth contributed to observed microbiome structure. This analysis showed that sequencing depth explained minimal

variation compared to country ($R^2 = 0.005$ vs. $R^2 = 0.28$). We have revised the manuscript to temper statements regarding standardization and to clarify that, while methodological harmonization reduces technical variability, residual technical effects cannot be fully excluded in the absence of mock community controls (pages 4 & 10).

Comment 2.3

"Line 111: Japan vs..?"

Response:

We thank the reviewer for pointing this out. We have ensured that no extra punctuation is observed here.

Comment 2.4

"Lines 107 – 130 and Figure 1:
a. One of the top 10 metadata features contributing to both PC1 and PC2 is Sex. And Table 1 shows that there were significant differences in study subject sex by country. Could the differences observed in cosmetics/supplement use by country be driven by sex differences in participants within countries?
b. The remaining top 10 metadata variables driving variation across PC1 are all BMI related. The methods describe great care that was taken to manage the high number of variables in this dataset. However, is it possible that some additional variable reduction regarding body weight/size could (should?) have taken place prior to performing this analysis."

Response:

We thank the reviewer for these important observations.
a. We agree that differences in sex distribution across countries could influence variables such as cosmetics and supplement use. To address this, we assessed whether cosmetics and vitamins B and D supplement use remained significantly different across countries within a female-only subset, and found that these differences persisted. In addition, we performed PERMANOVA analyses including sex and cosmetics frequency as covariates when assessing associations between country and microbiome composition. Geographic differences remained highly significant after adjustment, with country explaining substantially more variation than sex or cosmetics use (Results, page 5). These findings indicate that the observed differences are not driven by sex composition alone.
b. We thank the reviewer for this thoughtful comment and agree that additional variable reduction related to body weight and size is appropriate prior to dimensionality reduction. In response, we removed redundant measures (BMI categories, and obesity status) and repeated the analysis. The updated results are reflected in Figure 1e–f and the Results section (page 4).

Comment 2.5

"Lines 231 – 233: Fermented foods can be difficult to classify in food records. Can information on how and why the particular foods were included/excluded for the analysis shown in Figure S6 be added to the methods?"

Response:

We thank the reviewer for this important point. We agree that classification of fermented foods can vary depending on dietary assessment methods. In this study, analyses of fermented foods were restricted to the Japanese cohort, as *Bacillus subtilis* OGUs were predominantly observed in Japanese participants. Within this cohort, fermented food items were identified based on standardized food groupings derived from FFQ data. Foods were classified as fermented if they were produced through microbial fermentation and consistently annotated as such (e.g., natto, yogurt, kimchi, fermented soy products, and pickled vegetables). These categories were defined a priori based on established classifications in nutritional databases rather than selected based on observed associations. We have added additional details to the Methods section to clarify how fermented foods were defined and selected for analysis (page 12).

Comment 2.6:

"Line 245: Mash?"

Response:

We thank the reviewer for this comment. We have clarified the description of Mash in the revised manuscript (page 7). Specifically, we now define Mash as a MinHash-based approach for estimating genome-wide sequence similarity and include the appropriate citation (Ondov et al., 2016).

Comment 2.7:

"Line 418: What does the BCL acronym stand for?"

Response:

We thank the reviewer for bringing this to our attention. We have clarified the meaning of the acronym "BCL" in the revised manuscript by defining it as "binary base call" files (page 12).

Comment 2.8:

"Line 491: Reference for gemelli?"

Response:

We have clarified the use of gemelli in the revised manuscript by including the repository link (page 15).

Comment 2.9:

What was the rationale for artificially reducing variation within a given variable? For example, the home cooked meals frequency? It seems like retaining the variation in the number of home cooked meals reported by individuals would benefit the multivariate nature of the analysis performed.

Response:

We thank the reviewer for this important point. We agree that retaining the full range of variation in variables such as home-cooked meal frequency may, in principle, capture additional information.

However, variable reduction was performed to ensure consistency and statistical robustness across countries with substantially different distributions of metadata.

In particular, many variables (including home-cooked meal frequency) exhibited sparse or uneven category representation across cohorts. Retaining all categories resulted in small sample sizes within specific groups in certain countries, which can reduce statistical power and lead to unstable estimates in multivariate analyses. To address this, we collapsed categories into broader, harmonized groupings that ensured sufficient sample size within each category across all countries. Importantly, these variables were used primarily in exploratory analyses of metadata structure, and key conclusions were based on downstream models that do not rely on fine-grained categorization of these variables.

We have clarified this rationale in the Methods section (page 13).

Response to Reviewers

Title: The Human Diets & Microbiome Initiative: A five-country analysis of geographic and dietary drivers of gut microbiome composition and genomic variation

General Response

We thank the editor and reviewers for their decision to publish our manuscript. We have completed the Authors Checklist requested by the editor, and below we provide our response to the remaining reviewer comment.

Reviewer #1

“The revised manuscript is substantially improved and addresses the major concerns raised in the original review. The clarification of microbiome data representations, additional sequencing-depth analyses, expanded genome-resolved analyses, and more cautious interpretation significantly strengthen the work. I have only one remaining suggestion. While I appreciate the authors' justification for the ≥30 MAG threshold in terms of statistical power, I still believe that read mapping against representative MAGs or species-level genome bins would provide valuable complementary information regarding biological prevalence and would help contextualize the observed genomic differentiation. Aside from this point, I am satisfied with the revision.

Specific comment

- Biological prevalence versus MAG recovery.

I appreciate the authors' justification for the ≥30 MAG threshold in terms of statistical power for genome-resolved analyses. The explanation clarifies why this threshold is necessary for the downstream PERMANOVA and GWAS approaches. However, this does not fully address the original concern that MAG recovery is influenced by assembly success, sequencing depth, and genome characteristics, and therefore does not necessarily reflect the true biological prevalence of a species across populations. For this reason, I still believe that mapping metagenomic reads back to representative MAGs or species-level genome bins would provide valuable complementary information on species prevalence and abundance across cohorts. Such an analysis would not replace the genome-resolved approach, but rather complement it by providing an additional biological context for interpreting the observed genomic differentiation. This would help readers assess whether the strongest genomic signals are associated with widespread and abundant taxa or are restricted to a smaller subset of the microbiome.”

Response:

We thank the reviewer for this helpful suggestion. We agree that MAG recovery is influenced by assembly success, sequencing depth, and genome characteristics, and therefore should not be interpreted as a direct proxy for biological prevalence. To address this point, we performed an additional read-mapping analysis in which quality-controlled metagenomic reads were mapped back to the dereplicated MAG database used for the genome-resolved analyses. For each of the 73 species-level genome bins retained using the ≥30 MAG threshold, we summarized MAG recovery, breadth-based prevalence, and mapped-read abundance across the US, UK, Spain, Mexico, and Japan cohorts. Species detection was defined using a ≥40% genome-breadth threshold and ≥10× read depth, and abundance was summarized as the median percentage of mapped reads within each cohort.

This analysis is now included as Supplementary Fig. S8, and the Results and Methods sections have been amended accordingly. The new figure shows that many species retained for genome-resolved analyses are broadly detectable by read mapping, while also demonstrating that MAG recovery, breadth-based prevalence, and mapped-read abundance are related but not interchangeable. We therefore clarify that the ≥30 MAG threshold was used to ensure sufficient statistical power for genome-resolved PERMANOVA and GWAS analyses, rather than as a direct measure of species prevalence. We believe this complementary analysis provides the requested biological context for interpreting the observed genomic differentiation.
